# The minimal-ABC trees with *B_1_*-branches

**DOI:** 10.1371/journal.pone.0195153

**Published:** 2018-04-18

**Authors:** Darko Dimitrov, Zhibin Du, Carlos M. da Fonseca

**Affiliations:** 1 Hochschule für Technik und Wirtschaft Berlin, Wilhelminenhofstraße 75A, D-12459 Berlin, Germany; 2 School of Mathematics and Statistics, Zhaoqing University, Zhaoqing 526061, Guangdong, China; 3 University of Primorska, Department of Mathematics, Glagoljsaška 8, 6000 Koper, Slovenia; University of Michigan, UNITED STATES

## Abstract

The atom-bond connectivity index (or, for short, ABC index) is a molecular structure descriptor bridging chemistry to graph theory. It is probably the most studied topological index among all numerical parameters of a graph that characterize its topology. For a given graph *G* = (*V*, *E*), the ABC index of *G* is defined as $ABC\left(G\right)=\sum_{ij\in E}\sqrt{\left(d_{i}+d_{j}-2\right)/\left(d_{i}d_{j}\right)}$, where *d*_*i*_ denotes the degree of the vertex *i*, and *ij* is the edge incident to the vertices *i* and *j*. A combination of physicochemical and the ABC index properties are commonly used to foresee the bioactivity of different chemical composites. Additionally, the applicability of the ABC index in chemical thermodynamics and other areas of chemistry, such as in dendrimer nanostars, benzenoid systems, fluoranthene congeners, and phenylenes is well studied in the literature. While finding of the graphs with the greatest ABC-value is a straightforward assignment, the characterization of the tree(s) with minimal ABC index is a problem largely open and has recently given rise to numerous studies and conjectures. A *B*_1_-branch of a graph is a pendent path of order 2. In this paper, we provide an important step forward to the full characterization of these minimal trees. Namely, we show that a minimal-ABC tree contains neither 4 nor 3 *B*_1_-branches. The case when the number of *B*_1_-branches is 2 is also considered.

## Introduction

The atom-bond connectivity index, widely known as ABC index, of a graph is a thoroughly studied vertex-degree-based graph invariant both in chemistry and mathematical communities. For a given simple graph *G* = (*V*, *E*), let us denote by *d*_*u*_ the degree of vertex *u*, and *uv* the edge incident to the vertices *u* and *v*. The atom-bond connectivity index (or, simply, ABC index) is a vertex-degree-based graph topological index, which is a variation of the Randić graph-theoretic invariant [1], and is defined as

$$\begin{array}{c} ABC\left(G\right)=\sum_{uv\in E}f\left(d_{u},d_{v}\right)\;, \end{array}$$

where

$$\begin{array}{c} f\left(d\left(u\right),d\left(v\right)\right)=\sqrt{\frac{d_{u}+d_{v}-2}{d_{u}d_{v}}}\;. \end{array}$$

The relevance of the ABC index, in what we call today chemical graph theory, was first revealed two decades ago by Estrada, Torres, Rodríguez, and Gutman in [2]. They disclosed the importance of the ABC index as an analytical instrument for modeling thermodynamic properties of organic chemical compounds. Ten years later, Estrada [3] uncovered the significance of ABC index on the stability of branched alkanes, based on at that time a novel quantum-theory-like exposition. These studies were the trigger point for an uncountable number of papers on a new found area: chemical graph theory. Just to give two examples, in [4] it is proved that the ABC index of both benzenoid systems and fluoranthene congeners, consisting of two benzenoid fragments, depend exclusively on the number of vertices, hexagons and inlets. The author also characterized the extremal catacondensed benzenoid systems with the maximal and minimal ABC indices. The case of the phenylenes was considered by [5]. Another example of the importance of this topological descriptor can be seen on the calculation of the ABC index of an infinite class of nanostar dendrimers, artificially manufactured or synthesized molecule built up from branched units called monomers [6].

Many problems persist open, though. For example, it is known that the star of a given order has the maximal ABC index [7]. However for the trees with minimal ABC index, we are still far from a full characterization. For some further conjectures and partial results the reader is referred to [8–12]. More progress about minimal ABC trees can be found in [13–18].

A path *v*_0_*v*_1_⋯*v*_*r*_ in a graph *G* is said to be a pendent path of length *r*, where *d*_*v*_0__ ≥ 3, *d*_*v*_1__ = ⋯ = *d*_*v*_*r*−1__ = 2, and *d*_*v*_*r*__ = 1.

For the tree(s) with minimal ABC index, the length of its pendent paths is of crucial importance. In particular, the next lemma has become a key result in this area:

**Lemma 1** [11, 19] *If* *T* *is a tree with minimal ABC index, then every pendent path in* *T* *is of length* 2 *or* 3, *and there is at most one pendent path of length* 3 *in* *T*.

In [20], Wang defined the greedy trees, for a given degree sequence, as follows:

**Definition 1.** *Suppose that the degrees of the non-leaf vertices are given, the greedy tree is achieved by the following ‘greedy algorithm’*:

1. *Label the vertex with the largest degree as* *v* (*the root*);
2. *Label the neighbors of* *v* as *v*_1_, *v*_2_, …, *assign the largest degree available to them such that* *d*(*v*_1_) ≥ *d*(*v*_2_) ≥ ⋯;
3. *Label the neighbors of* *v*_1_ (*except* *v*) *as* *v*_11_, *v*_12_, …, *such that they take all the largest degrees available and that* *d*(*v*_11_) ≥ *d*(*v*_12_) ≥ ⋯, *then do the same for* *v*_2_, *v*_3_, …;
4. *Repeat* (3) *for all newly labeled vertices, always starting with the neighbors of the labeled vertex with largest degree whose neighbors are not labeled yet*.

In particular, the vertex *i* is said to be the root of *T*, which is also the vertex lying on the first layer of *T*; the vertices *i*_1_, *i*_2_, … are said to be the vertices lying on the second layer of *T*; the vertices *i*_11_, *i*_12_, … are said to be the vertices lying on the third layer of *T*, and so on.

A major result attesting the importance of the greedy trees is the next proposition.

**Proposition 2** ([21, 22]). *Given the degree sequence, the greedy tree minimizes the ABC index*.

**Fig 1:**
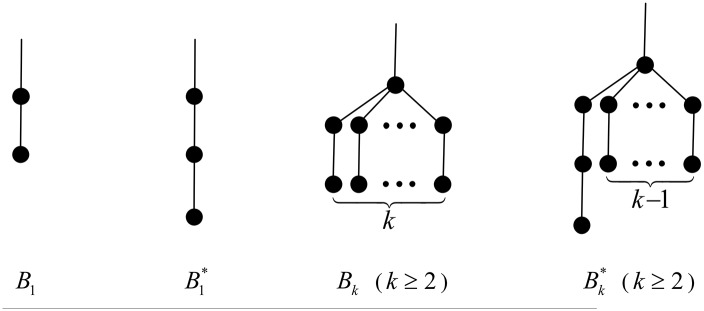
The *B*_*k*_- and $B_{k}^{*}$-branches for *k* ≥ 1.

From the previous considerations, different types of branches will play a crucial role in our quest. Namely, the *B*_*k*_-branches, with *k* ≥ 1, and the $B_{k}^{*}$-branches, with *k* ≥ 1, are illustrated in Fig 1.

In this regard, the most relevant results on minimal-ABC trees are listed next.

**Proposition 3** ([23, Theorem 3.2]). *A minimal-ABC tree does not contain* *B*_*k*_-*branch, with* *k* > 4.

**Proposition 4** ([24, Proposition 3.4]). *A minimal-ABC tree does not contain a* *B*_3_-*branch and a* $B_{1}^{*}$-*branch sharing a common parent vertex*.

**Proposition 5** ([23, Lemma 3.3(a)]). *A minimal-ABC tree does not contain a* *B*_4_-*branch and a* *B*_1_-*branch sharing a common parent vertex*.

**Proposition 6** ([25, Theorem 3.4]). *A minimal-ABC tree of order* *n* > 18 *with a pendent path of length 3 may contain a* *B*_2_-*branch if and only if it is of order* 161 *or* 168. *Moreover, in this case, a minimal-ABC tree is comprised of a single central vertex*, *B*_3_-*branches and one* *B*_2_, *including a pendent path of length* 3 *that may belong to a* $B_{3}^{*}$-branch or $B_{2}^{*}$-*branch*.

As a consequence of Proposition 6, we get the following proposition immediately.

**Proposition 7**. *A minimal-ABC tree cannot contain a* *B*_2_-*branch and a* $B_{1}^{*}$-*branch simultaneously*.

Recently, the authors were able to show in [26] that a minimal-ABC tree cannot contain simultaneously a *B*_4_-branch and *B*_1_- or *B*_2_-branches.

Recall that a *k*-*terminal vertex* of a rooted tree is a vertex of degree *k* + 1 ≥ 3, which is a parent of only *B*_≥1_-branches, such that at least one branch among them is a *B*_1_-branch (or $B_{1}^{*}$-branch). The (sub)tree, induced by a *k*-*terminal vertex* and all its (direct and indirect) children (descendant) vertices, is called a *k*-*terminal branch* or *T*_*k*_-*branch*.

**Proposition 8** ([27, Proposition 2.13]). *A minimal-ABC tree contains at most one* *T*_*k*_-*branch, with* *k* ≥ 2.

**Proposition 9** ([27, Theorem 3.5]). *A minimal-ABC tree contains at most four* *B*_1_-*branches*.

Although all the progress that has been lately made, the minimal-ABC trees seem still far from a full characterization. This paper contributes for this task. Specifically, we show that such trees contain neither 4 nor 3 *B*_1_-branches. The case when we have 2 *B*_1_-branches is also considered in the last section.

## Preliminaries and methods

### Lemmas

First we recall some technical lemmas.

**Lemma 10** ([23, Proposition A.3]). *Let*

$$\begin{array}{c} g(x,y)=f(x+\Delta x,y-\Delta y)-f(x,y) \end{array}$$

*with real numbers* *x*, *y* ≥ 2, Δ*x* ≥ 0, 0 ≤ Δ*y* < *y*. *Then* *g*(*x*, *y*) *increases in* *x* *and decreases in* *y*.

Due to the symmetry of the function *g*(*x*, *y*), we can also get an equivalent version of Lemma 10.

**Lemma 11**. *Let*

$$\begin{array}{c} g(x,y)=f(x-\Delta x,y+\Delta y)-f(x,y) \end{array}$$

*with real numbers* *x*, *y* ≥ 2, 0 ≤ Δ*x* < *x*, Δ*y* ≥ 0. *Then* *g*(*x*, *y*) *decreases in* *x* *and increases in* *y*.

In a similar fashion we have:

**Lemma 12**. *Let* *h*(*x*, *y*) = (*y* − 4)*f*(*x* + *y* − 5, 4) − *f*(*x*, *y*), *where* *x* ≥ *y* *and* *y* = 6, 7, 8, 9, 10, 11. *Then for every fixed* *y*, *the function* *h*(*x*, *y*) *decreases in* *x* ≥ *y*.

*proof.* We only prove the case when *y* = 6. The other cases are similar.

Suppose that *y* = 6. Then *h*(*x*, 6) = 2*f*(*x* + 1, 4) − *f*(*x*, 6).

First we have

$$\begin{array}{c} \sqrt{6}x^{2}{\left(x+1\right)}^{\frac{3}{2}}\sqrt{\left(x+3)(x+4\right)}h^{\prime }\left(x,6\right)=2{\left(x+1\right)}^{\frac{3}{2}}\sqrt{x(x+3)}-\sqrt{6}x^{2}\sqrt{x+4}\;. \end{array}$$

Next, it is readily verified that

$$\begin{array}{c} 2{\left(x+1\right)}^{\frac{3}{2}}\sqrt{x(x+3)}-\sqrt{6}x^{2}\sqrt{x+4}<0 \end{array}$$

for *x* ≥ 6.

Now it follows that *h*′(*x*, 6) < 0, i.e., *h*(*x*, 6) decreases in *x* ≥ 6.

Similar to the proof of Lemma 12, we can also get the following lemma.

**Lemma 13**. *Let* *ℓ*(*x*, *y*) = (*y* − 3)*f*(*x* + *y* − 4, 3) − *f*(*x*, *y*), *where* *y* = 5, 7, 8, 9.

1. *When* *y* = 5, *the function* *ℓ*(*x*, 5) *increases in* *x* > 0.
2. *When* *y* = 7, *the function* *ℓ*(*x*, 7) *decreases in* *x* ≥ 19.
3. *When* *y* = 8, *the function* *ℓ*(*x*, 8) *decreases in* *x* ≥ 17.
4. *When* *y* = 9, *the function* *ℓ*(*x*,9) *decreases in* *x* ≥ 16.

### The root of *B*_1_-branches

A Kragujevac tree is a tree comprising of a single central vertex, *B*_*k*_-branches, with *k* ≥ 1, and at most one $B_{k}^{*}$-branch.

**Lemma 14** ([28, Theorem 11]). *If* *T* *is a Kragujevac tree with minimal ABC index, and the degree of the central vertex of* *T* *is at least* 19, *then* *T* *contains no* *B*_1_-*branch*.

Taking into account Lemma 14, we can establish the main result in this section.

**Proposition 15**. *If* *T* *is a minimal-ABC tree on more than* 122 *vertices containing* *B*_1_-*branches, then the* *B*_1_-*branches cannot be attached to the root vertex of* *T*.

*proof.* Observe that the *B*_1_-branches of *T* are attached to the same vertex, say *u*, otherwise, there are at least two *T*_*k*_-branches, which is a contradiction to Proposition 8. Suppose to the contrary that *u* is the root vertex of *T*.

**Fig 2:**
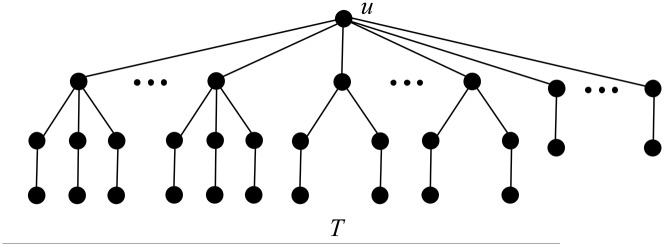
The tree *T* in the proof of Proposition 15.

First, by Proposition 3, *u* contains no *B*_*k*_-branch with *k* > 4. Next by Proposition 5, *u* contains no *B*_4_-branch, and by Propositions 4 and 7, *u* contains no $B_{1}^{*}$-branch, no matter *u* has *B*_3_-branches or *B*_2_-branches. Now we may deduce that the branches attached to *u* must be *B*_3_-, *B*_2_- or *B*_1_-branches, i.e., *T* is of the structure as depicted in Fig 2.

Notice that *T* is actually a Kragujevac tree. Denote by *d*_*u*_ the degree of *u* in *T*.

If *d*_*u*_ ≥ 19, then from Lemma 14, *T* contains no *B*_1_-branch, which is a contradiction to the assumption for the existence of *B*_1_-branches in *T*.

If *d*_*u*_ ≤ 18, then recall that every branch attached to *u* in *T* is a *B*_*k*_-branch with *k* = 1, 2, 3, and thus the order of *T* is at most

$$\begin{array}{c} 1+7\left(d_{u}-1\right)+2=7d_{u}-4\leq 122\;, \end{array}$$

which is a contradiction to the assumption for the order of *T*.

Now the result follows.

Since all the minimal-ABC trees of order up to 300 are completely determined in [29], we may assume that the trees considered in our main results have more than 300 vertices.

### Switching transformation

Before we proceed with the main results of this paper, we present the so-called *switching transformation* explicitly stated by Lin, Gao, Chen, and Lin [30].

**Lemma 16** (Switching transformation). *Let* *G* = (*V*, *E*) *be a connected graph with* *uv*, *xy* ∈ *E*(*G*) *and* *uy*, *xv* ∉ *E*(*G*). *Let* *G*_1_ = *G* − *uv* − *xy* + *uy* + *xv*. *If* *d*(*u*) ≥ *d*(*x*) *and* *d*(*v*) ≤ *d*(*y*), *then* *ABC*(*G*_1_) ≤ *ABC*(*G*), *with the equality if and only if* *d*(*u*) = *d*(*x*) or *d*(*v*) = *d*(*y*).

The switching transformation was used in the proofs of some characterizations of the minimal-ABC trees, and the following observation that will be applied in the further analysis.

**Observation 1**. *Let* *G* *be a minimal-ABC tree with the root vertex* *v*_0_ *and let* *v*_0_, *v*_1_, …, *v*_*n*_ *be the sequence of vertices obtain by the breadth-first search of* *G*. *If* *d*(*v*_*i*_), *d*(*v*_*j*_) ≥ 3 *and* *i* < *j*, *then by Lemma* 16, *we may assume that* *d*(*v*_*i*_) ≥ *d*(*v*_*j*_).

From Observation 1, we may assume that the trees considered are all greedy trees.

## Results

### The existence of four *B*_1_-branches

In this section we will prove our first main result: Any minimal-ABC tree cannot contain four *B*_1_-branches.

The following result is recent and establishes a forbidden configuration for minimal-ABC trees.

**Fig 3:**
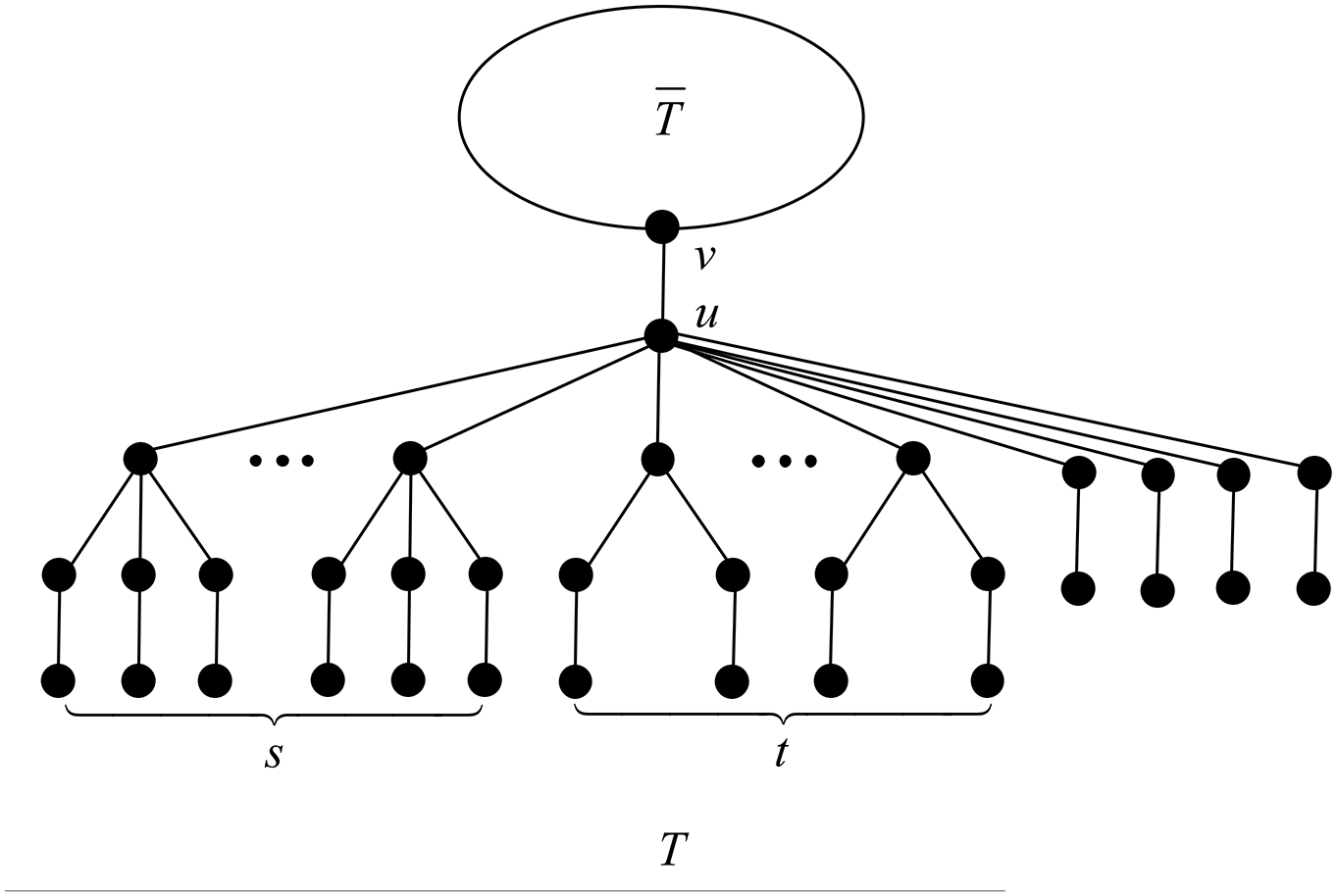
The tree *T* in Proposition 17 and Theorem 18.

**Proposition 17** ([27, Proposition 3.2]). *When* *s* + *t* > 6, *the configuration* *T* *depicted in* Fig 3 *cannot occur in a minimal-ABC tree*.

We are ready now to state the main result of this section.

**Theorem 18**. *A minimal-ABC tree cannot contain four* *B*_1_-*branches*.

*proof.* Suppose to the contrary that *T* is a minimal-ABC tree containing exactly four *B*_1_-branches. Observe that the four *B*_1_-branches are attached to the same vertex, say *u*, otherwise, there are at least two *T*_*k*_-branches, which is a contradiction to Proposition 8. Moreover, by Proposition 15, *u* is not the root vertex of *T*. Let us denote by *v* the parent of *u*.

First, by Proposition 3, *u* contains no *B*_*k*_-branch with *k* > 4. Next by Proposition 5, *u* contains no *B*_4_-branch, and by Propositions 4 and 7, *u* contains no $B_{1}^{*}$-branch, no matter *u* has *B*_3_-branches or *B*_2_-branches. Now we may deduce that the branches attached to *u* must be *B*_3_-, *B*_2_- or *B*_1_-branches, i.e., *T* is of the structure depicted in Fig 3.

Denote by *s* the number of *B*_3_-branches attached to *u*, and *t* the number of *B*_2_-branches attached to *u*. Clearly, *s* + *t* ≥ 1, and *s* + *t* ≤ 6 from Proposition 17.

Let *d*_*x*_ be the degree of vertex *x* in *T*.

Observe that *d*_*v*_ ≥ *d*_*u*_ = *s* + *t* + 5 from Proposition 2.

**Case 1.** *t* = 0.

**Fig 4:**
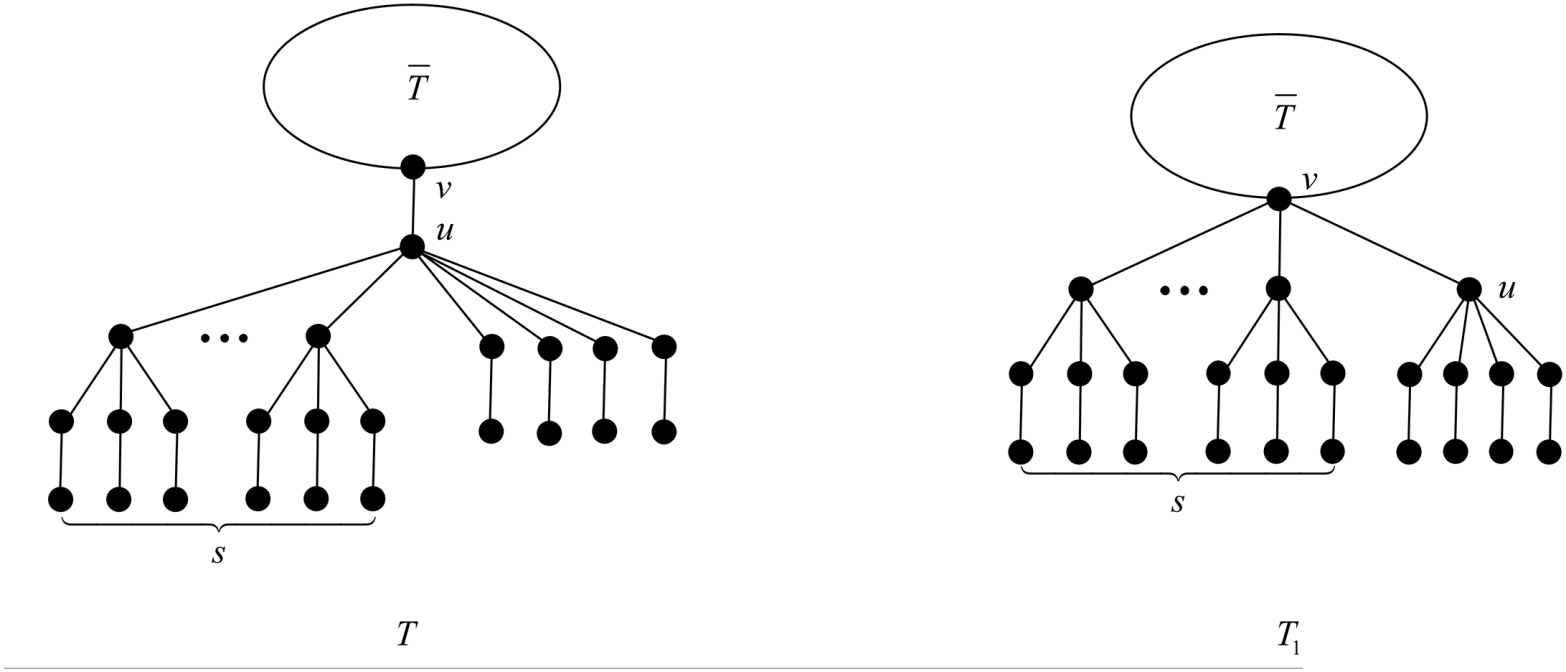
The transformation $T_{1}$ in Case 1 of Theorem 18.

In this case, we apply the transformation $T_{1}$ depicted in Fig 4.

After applying $T_{1}$, the degree of vertex *v* increases by *s*, while the degree of vertex *u* decreases by *s*. The rest of the vertices do not change their degrees. The change of the ABC index after applying $T_{1}$ is

$$\begin{array}{ccc} ABC\left(T_{1}\right)-ABC\left(T\right) & = & \sum_{xv\in E(\bar{T})}\left(f\left(d_{v}+s,d_{x}\right)-f\left(d_{v},d_{x}\right)\right)\\ & & +f\left(d_{v}+s,5\right)-f\left(d_{v},s+5\right)+s\left(f\left(d_{v}+s,4\right)-f\left(s+5,4\right)\right)\;. \end{array}$$

Clearly, *f*(*d*_*v*_ + *s*, *d*_*x*_) − *f*(*d*_*v*_, *d*_*x*_) < 0 for $xv\in E(\bar{T})$, and thus

$$\begin{array}{ccc} ABC\left(T_{1}\right)-ABC\left(T\right) & < & f\left(d_{v}+s,5\right)-f\left(d_{v},s+5\right)+s\left[f\left(d_{v}+s,4\right)-f\left(s+5,4\right)\right]\\ & = & \left(s+1\right)f\left(d_{v}+s,4\right)-f\left(d_{v},s+5\right)\\ & & +f\left(d_{v}+s,5\right)-f\left(d_{v}+s,4\right)-s\cdot f\left(s+5,4\right)\;. \end{array}$$

Recall that *d*_*v*_ ≥ *s* + 5 from Proposition 2. On one hand, by Lemma 12, (*s* + 1)*f*(*d*_*v*_ + *s*, 4) − *f*(*d*_*v*_, *s* + 5) decreases in *d*_*v*_ ≥ *s* + 5. On the other hand, by Lemma 11, *f*(*d*_*v*_ + *s*, 5) − *f*(*d*_*v*_ + *s*, 4) also decreases in *d*_*v*_ ≥ *s* + 5. So we get that

$$\begin{array}{ccc} ABC\left(T_{1}\right)-ABC\left(T\right) & < & \left(s+1)f((s+5)+s,4)-f(s+5,s+5\right)\\ & & +f((s+5)+s,5)-f((s+5)+s,4)-s\cdot f(s+5,4)\\ & = & \left(s+1)f(2s+5,4)-f(s+5,s+5\right)\\ & & +f(2s+5,5)-f(2s+5,4)-s\cdot f(s+5,4)\;. \end{array}$$ (1)

By virtue of Mathematica, the right-hand side of (1) is negative, equivalently *ABC*(*T*_1_) < *ABC*(*T*), follows from direct calculation, for 1 ≤ *s* ≤ 6.

**Case 2.** *t* ≥ 1.

**Fig 5:**
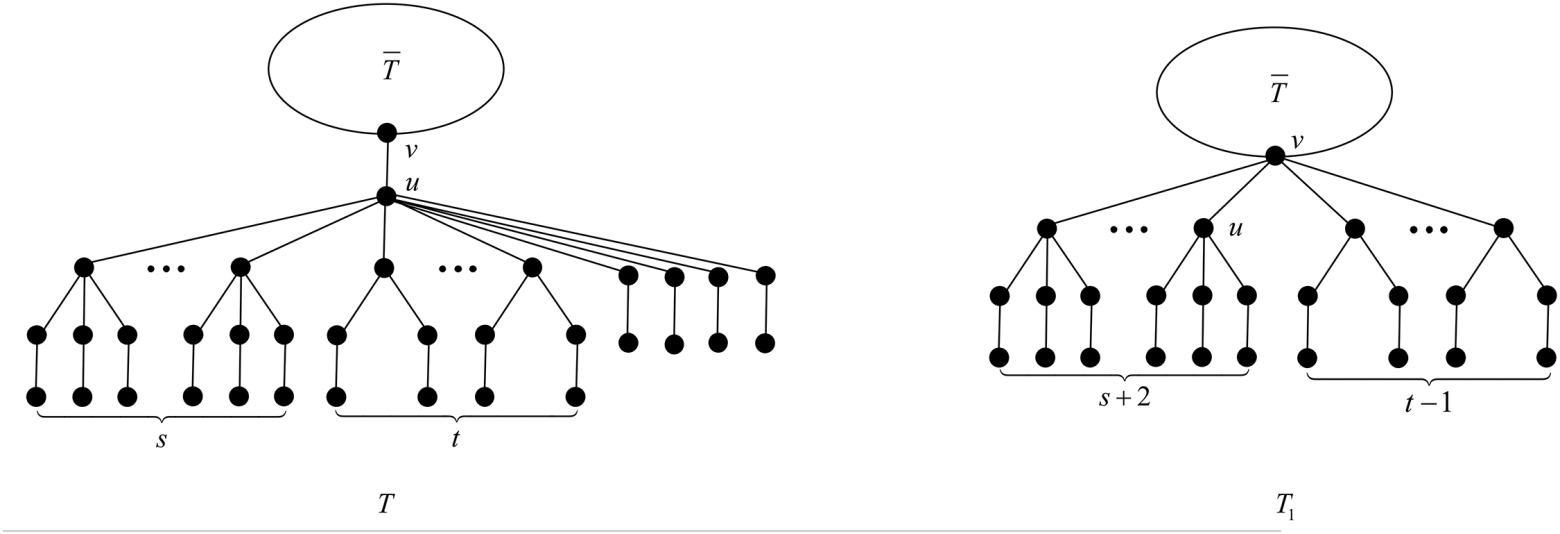
The transformation $T_{2}$ in Case 2 of Theorem 18.

In this case, we apply the transformation $T_{2}$ depicted in Fig 5.

After applying $T_{2}$, the degree of vertex *v* increases by *s* + *t*, while the degree of vertex *u* decreases to 4, and a child of *u* in *T* belonging to a *B*_2_-branch increases its degree from 3 to 4. The rest of the vertices do not change their degrees. The change of the ABC index after applying $T_{2}$ is

$$\begin{array}{ccc} ABC\left(T_{1}\right)-ABC\left(T\right) & = & \sum_{xv\in E(\bar{T})}\left(f\left(d_{v}+s+t,d_{x}\right)-f\left(d_{v},d_{x}\right)\right)\\ & & +s(f\left(d_{v}+s+t,4\right)-f\left(s+t+5,4\right))\\ & & +\left(t-1\right)(f\left(d_{v}+s+t,3\right)-f\left(s+t+5,3\right))\\ & & +2f\left(d_{v}+s+t,4\right)-f\left(s+t+5,3\right)\\ & & -f(d_{v},s+t+5)\;. \end{array}$$ (2)

Clearly, *f*(*d*_*v*_ + *s* + *t*, *d*_*x*_) − *f*(*d*_*v*_, *d*_*x*_) < 0 for $xv\in E(\bar{T})$, and thus

$$\begin{array}{ccc} ABC\left(T_{1}\right)-ABC\left(T\right) & < & s(f\left(d_{v}+s+t,4\right)-f\left(s+t+5,4\right))\\ & & +\left(t-1\right)(f\left(d_{v}+s+t,3\right)-f\left(s+t+5,3\right))\\ & & +2f\left(d_{v}+s+t,4\right)-f\left(s+t+5,3\right)-f\left(d_{v},s+t+5\right)\;. \end{array}$$

Let *r* = *s* + *t* be a fixed number. Recall that 1 ≤ *r* ≤ 6.

Now we have

$$\begin{array}{ccc} ABC\left(T_{1}\right)-ABC\left(T\right) & < & \left(r-t\right)(f\left(d_{v}+r,4\right)-f\left(r+5,4\right))\\ & & +\left(t-1\right)(f\left(d_{v}+r,3\right)-f\left(r+5,3\right))\\ & & +2f\left(d_{v}+r,4\right)-f\left(r+5,3\right)-f\left(d_{v},r+5\right)\;. \end{array}$$ (3)

For the right-hand side of (3), notice that the coefficient of *t* is

$$\begin{array}{c} \left(f\left(d_{v}+r,3\right)-f\left(r+5,3\right)\right)-\left(f\left(d_{v}+r,4\right)-f\left(r+5,4\right)\right)\;. \end{array}$$

Since *d*_*v*_ > 5, from Lemma 10, *f*(*d*_*v*_ + *r*, *y*) − *f*(*r* + 5, *y*) decreases in *y* ≥ 2, thus we may deduce that

$$\begin{array}{c} \left(f\left(d_{v}+r,3\right)-f\left(r+5,3\right)\right)-\left(f\left(d_{v}+r,4\right)-f\left(r+5,4\right)\right)>0\;. \end{array}$$

Together with *t* ≤ *r*, we have

$$\begin{array}{ccc} ABC\left(T_{1}\right)-ABC\left(T\right) & < & \left(r-r\right)(f\left(d_{v}+r,4\right)-f\left(r+5,4\right))\\ & & +\left(r-1\right)(f\left(d_{v}+r,3\right)-f\left(r+5,3\right))\\ & & +2f\left(d_{v}+r,4\right)-f\left(r+5,3\right)-f\left(d_{v},r+5\right)\\ & = & \left(r-1\right)f\left(d_{v}+r,3\right)+2f\left(d_{v}+r,4\right)\\ & & -f\left(d_{v},r+5\right)-r\cdot f\left(r+5,3\right)\;. \end{array}$$ (4)

Recall that *d*_*v*_ ≥ *r* + 5 from Proposition 2.

**Subcase 2.1.** *r* = 1.

If *r* = 1, then by (4), we have

$$\begin{array}{c} ABC\left(T_{1}\right)-ABC\left(T\right)<2f\left(d_{v}+1,4\right)-f\left(d_{v},6\right)-f\left(6,3\right)\;. \end{array}$$

Moreover, by Lemma 12, we know that 2*f*(*d*_*v*_ + 1, 4) − *f*(*d*_*v*_, 6) decreases in *d*_*v*_ ≥ 6, thus

$$\begin{array}{c} ABC\left(T_{1}\right)-ABC\left(T\right)<2f\left(6+1,4\right)-f\left(6,6\right)-f\left(6,3\right)<0\;, \end{array}$$

i.e., *ABC*(*T*_1_) < *ABC*(*T*).

**Subcase 2.2.** *r* = 2.

If *r* = 2, then by (4), we have

$$\begin{array}{ccc} ABC\left(T_{1}\right)-ABC\left(T\right) & < & f\left(d_{v}+2,3\right)+2f\left(d_{v}+2,4\right)-f\left(d_{v},7\right)-2f\left(7,3\right)\\ & = & 3f\left(d_{v}+2,4\right)-f\left(d_{v},7\right)\\ & & +f\left(d_{v}+2,3\right)-f\left(d_{v}+2,4\right)-2f\left(7,3\right)\;. \end{array}$$

Moreover, by Lemma 12, we know that 3*f*(*d*_*v*_ + 2, 4) − *f*(*d*_*v*_, 7) decreases in *d*_*v*_ ≥ 7, and by Lemma 10, *f*(*d*_*v*_ + 2, 3) − *f*(*d*_*v*_ + 2, 4) increases in *d*_*v*_, thus

$$\begin{array}{c} f\left(d_{v}+2,3\right)-f\left(d_{v}+2,4\right)\leq \lim_{d_{v}\to +\infty }\left(f\left(d_{v}+2,3\right)-f\left(d_{v}+2,4\right)\right)=\sqrt{\frac{1}{3}}-\sqrt{\frac{1}{4}}\;. \end{array}$$

So for *d*_*v*_ ≥ 11, we get that

$$\begin{array}{c} ABC\left(T_{1}\right)-ABC\left(T\right)<3f\left(11+2,4\right)-f\left(11,7\right)+\sqrt{\frac{1}{3}}-\sqrt{\frac{1}{4}}-2f\left(7,3\right)<0\;, \end{array}$$

i.e., *ABC*(*T*_1_) < *ABC*(*T*). For the remaining cases that 7 ≤ *d*_*v*_ ≤ 10, by virtue of Mathematica, the right-hand side of (4) is negative, equivalently *ABC*(*T*_1_) < *ABC*(*T*), follows from direct calculation easily.

**Subcase 2.3.** *r* = 3.

If *r* = 3, then by (4), we have

$$\begin{array}{ccc} ABC\left(T_{1}\right)-ABC\left(T\right) & < & 2f\left(d_{v}+3,3\right)+2f\left(d_{v}+3,4\right)-f\left(d_{v},8\right)-3f\left(8,3\right)\\ & = & 4f\left(d_{v}+3,4\right)-f\left(d_{v},8\right)\\ & & +2\left(f\left(d_{v}+3,3\right)-f\left(d_{v}+3,4\right)\right)-3f\left(8,3\right)\;. \end{array}$$

Moreover, by Lemma 12, we know that 4*f*(*d*_*v*_ + 3, 4) − *f*(*d*_*v*_, 8) decreases in *d*_*v*_ ≥ 8, and by Lemma 10, *f*(*d*_*v*_ + 3, 3) − *f*(*d*_*v*_ + 3, 4) increases in *d*_*v*_, thus

$$\begin{array}{c} f\left(d_{v}+3,3\right)-f\left(d_{v}+3,4\right)\leq \lim_{d_{v}\to +\infty }\left(f\left(d_{v}+3,3\right)-f\left(d_{v}+3,4\right)\right)=\sqrt{\frac{1}{3}}-\sqrt{\frac{1}{4}}\;. \end{array}$$

So for *d*_*v*_ ≥ 20, we get that

$$\begin{array}{c} ABC\left(T_{1}\right)-ABC\left(T\right)<4f\left(20+3,4\right)-f\left(20,8\right)+2\left(\sqrt{\frac{1}{3}}-\sqrt{\frac{1}{4}}\right)-3f\left(8,3\right)<0\;, \end{array}$$

i.e., *ABC*(*T*_1_) < *ABC*(*T*). For the remaining cases that 8 ≤ *d*_*v*_ ≤ 19, by virtue of Mathematica, the right-hand side of (4) is negative, equivalently *ABC*(*T*_1_) < *ABC*(*T*), follows from direct calculation easily.

**Subcase 2.4.** *r* = 4.

If *r* = 4, then by (4), we have

$$\begin{array}{ccc} ABC\left(T_{1}\right)-ABC\left(T\right) & < & 3f\left(d_{v}+4,3\right)+2f\left(d_{v}+4,4\right)-f\left(d_{v},9\right)-4f\left(9,3\right)\\ & = & 5f\left(d_{v}+4,4\right)-f\left(d_{v},9\right)\\ & & +3\left(f\left(d_{v}+4,3\right)-f\left(d_{v}+4,4\right)\right)-4f\left(9,3\right)\;. \end{array}$$

Moreover, by Lemma 12, we know that 5*f*(*d*_*v*_ + 4, 4) − *f*(*d*_*v*_, 9) decreases in *d*_*v*_ ≥ 9, and by Lemma 10, *f*(*d*_*v*_ + 4, 3) − *f*(*d*_*v*_ + 4, 4) increases in *d*_*v*_, thus

$$\begin{array}{c} f\left(d_{v}+4,3\right)-f\left(d_{v}+4,4\right)\leq \lim_{d_{v}\to +\infty }\left(f\left(d_{v}+4,3\right)-f\left(d_{v}+4,4\right)\right)=\sqrt{\frac{1}{3}}-\sqrt{\frac{1}{4}}\;. \end{array}$$

So for *d*_*v*_ ≥ 31, we get that

$$\begin{array}{c} ABC\left(T_{1}\right)-ABC\left(T\right)<5f\left(31+4,4\right)-f\left(31,9\right)+3\left(\sqrt{\frac{1}{3}}-\sqrt{\frac{1}{4}}\right)-4f\left(9,3\right)<0\;, \end{array}$$

i.e., *ABC*(*T*_1_) < *ABC*(*T*). For the remaining cases that 9 ≤ *d*_*v*_ ≤ 30, by virtue of Mathematica, the right-hand side of (4) is negative, equivalently *ABC*(*T*_1_) < *ABC*(*T*), follows from direct calculation easily.

**Subcase 2.5.** *r* = 5.

If *r* = 5, then by (4), we have

$$\begin{array}{ccc} ABC\left(T_{1}\right)-ABC\left(T\right) & < & 4f\left(d_{v}+5,3\right)+2f\left(d_{v}+5,4\right)-f\left(d_{v},10\right)-5f\left(10,3\right)\\ & = & 6f\left(d_{v}+5,4\right)-f\left(d_{v},10\right)\\ & & +4\left(f\left(d_{v}+5,3\right)-f\left(d_{v}+5,4\right)\right)-5f\left(10,3\right)\;. \end{array}$$

Moreover, by Lemma 12, we know that 6*f*(*d*_*v*_ + 5, 4) − *f*(*d*_*v*_, 10) decreases in *d*_*v*_ ≥ 10, and by Lemma 10, *f*(*d*_*v*_ + 5, 3) − *f*(*d*_*v*_ + 5, 4) increases in *d*_*v*_, thus

$$\begin{array}{c} f\left(d_{v}+5,3\right)-f\left(d_{v}+5,4\right)\leq \lim_{d_{v}\to +\infty }\left(f\left(d_{v}+5,3\right)-f\left(d_{v}+5,4\right)\right)=\sqrt{\frac{1}{3}}-\sqrt{\frac{1}{4}}\;. \end{array}$$

So for *d*_*v*_ ≥ 42, we get that

$$\begin{array}{c} ABC\left(T_{1}\right)-ABC\left(T\right)<6f\left(42+5,4\right)-f\left(42,10\right)+4\left(\sqrt{\frac{1}{3}}-\sqrt{\frac{1}{4}}\right)-5f\left(10,3\right)<0\;, \end{array}$$

i.e., *ABC*(*T*_1_) < *ABC*(*T*). For the remaining cases that 10 ≤ *d*_*v*_ ≤ 41, by virtue of Mathematica, the right-hand side of (4) is negative, equivalently *ABC*(*T*_1_) < *ABC*(*T*), follows from direct calculation easily.

**Subcase 2.6.** *r* = 6.

If *r* = 6, then by (4), we have

$$\begin{array}{ccc} ABC\left(T_{1}\right)-ABC\left(T\right) & < & 5f\left(d_{v}+6,3\right)+2f\left(d_{v}+6,4\right)-f\left(d_{v},11\right)-6f\left(11,3\right)\\ & = & 7f\left(d_{v}+6,4\right)-f\left(d_{v},11\right)+5\left(f\left(d_{v}+6,3\right)-f\left(d_{v}+6,4\right)\right)\\ & & -6f(11,3)\;. \end{array}$$

Moreover, by Lemma 12, we know that 7*f*(*d*_*v*_ + 6, 4) − *f*(*d*_*v*_, 11) decreases in *d*_*v*_ ≥ 11, and by Lemma 10, *f*(*d*_*v*_ + 6, 3) − *f*(*d*_*v*_ + 6, 4) increases in *d*_*v*_, thus

$$\begin{array}{c} f\left(d_{v}+6,3\right)-f\left(d_{v}+6,4\right)\leq \lim_{d_{v}\to +\infty }\left(f\left(d_{v}+6,3\right)-f\left(d_{v}+6,4\right)\right)=\sqrt{\frac{1}{3}}-\sqrt{\frac{1}{4}}\;. \end{array}$$

So for *d*_*v*_ ≥ 56, we get that

$$\begin{array}{c} ABC\left(T_{1}\right)-ABC\left(T\right)<7f\left(56+6,4\right)-f\left(56,11\right)+5\left(\sqrt{\frac{1}{3}}-\sqrt{\frac{1}{4}}\right)-6f\left(11,3\right)<0\;, \end{array}$$

i.e., *ABC*(*T*_1_) < *ABC*(*T*). For the cases that 18 ≤ *d*_*v*_ ≤ 55, by virtue of Mathematica, the right-hand side of (4) is negative, equivalently *ABC*(*T*_1_) < *ABC*(*T*), follows from direct calculation easily.

As to the remaining cases that 11 ≤ *d*_*v*_ ≤ 17, let us be a bit more precisely in (2) about the term

$$\begin{array}{c} \sum_{xv\in E(\bar{T})}\left(f\left(d_{v}+s+t,d_{x}\right)-f\left(d_{v},d_{x}\right)\right)=\sum_{xv\in E(\bar{T})}\left(f\left(d_{v}+6,d_{x}\right)-f\left(d_{v},d_{x}\right)\right)\;. \end{array}$$

Notice that the degree of every neighbor of *v* in $\bar{T}$ is at least 3 from Proposition 2. Furthermore, by Lemma 10, *f*(*d*_*v*_ + 6, *d*_*x*_) − *f*(*d*_*v*_, *d*_*x*_) decreases in *d*_*x*_ ≥ 3, we may deduce that

$$\begin{array}{c} \sum_{xv\in E(\bar{T})}\left(f\left(d_{v}+6,d_{x}\right)-f\left(d_{v},d_{x}\right)\right)<f\left(d_{v}+6,3\right)-f\left(d_{v},3\right)\;. \end{array}$$

Now together with (4), it follows that

$$\begin{array}{ccc} ABC\left(T_{1}\right)-ABC\left(T\right) & < & f\left(d_{v}+6,3\right)-f\left(d_{v},3\right)\\ & & +5f\left(d_{v}+6,3\right)+2f\left(d_{v}+6,4\right)\\ & & -f\left(d_{v},11\right)-6f\left(11,3\right)\;. \end{array}$$ (5)

By virtue of Mathematica, the right-hand side of (5) is negative, equivalently *ABC*(*T*_1_) < *ABC*(*T*), for 11 ≤ *d*_*v*_ ≤ 17, follows from direct calculation easily.

Combining the above cases, the result follows easily.

### The existence of three *B*_1_-branches

We proceed proving in this section that a minimal-ABC tree does not contain three *B*_1_-branches. Before that, we consider some preliminary results.

**Fig 6:**
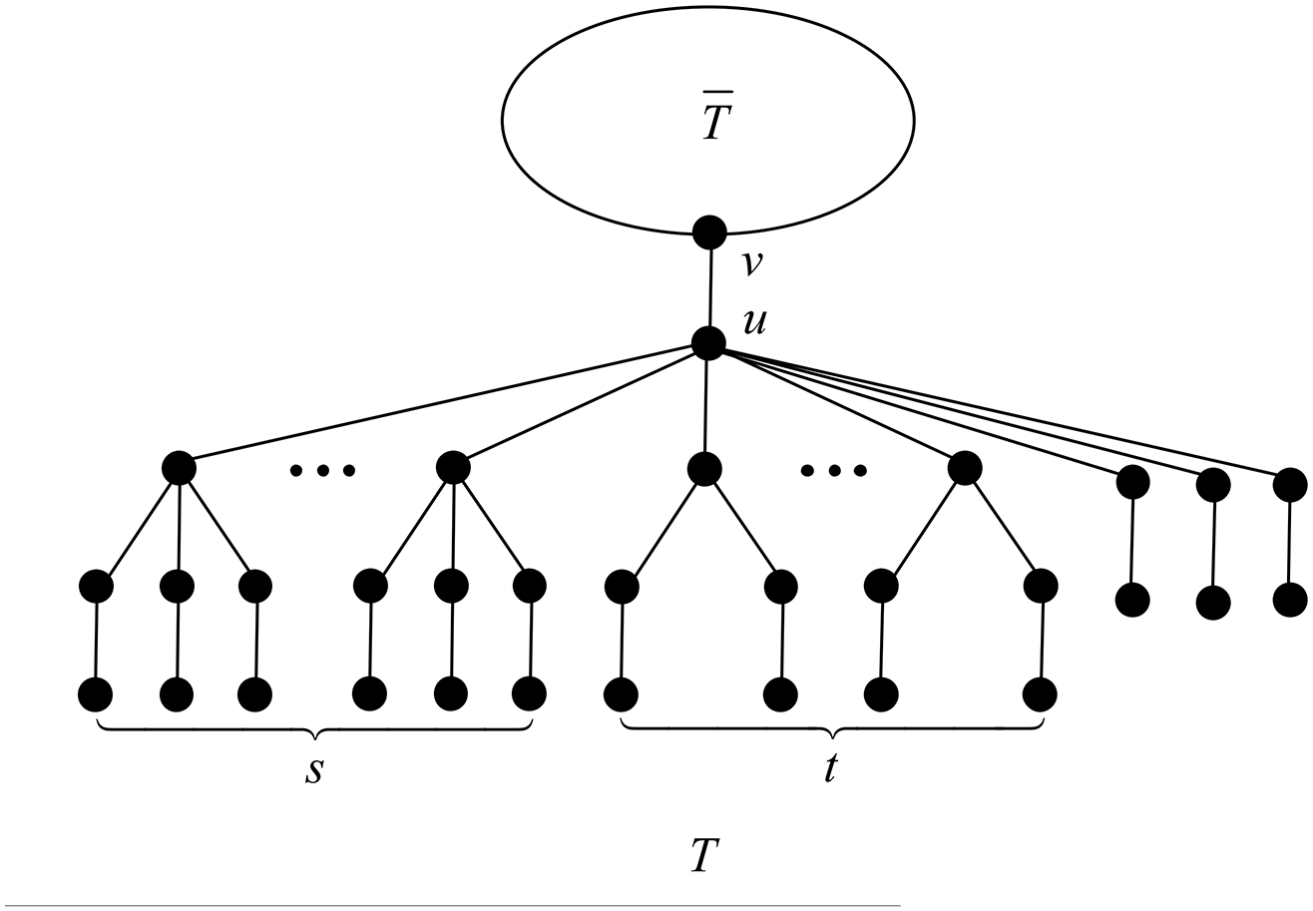
The tree *T* in Propositions 19, 20 and 21, and Theorem 22.

**Proposition 19** ([27, Proposition 3.2]). *When* *s* + *t* > 8, *the configuration* *T* *depicted in* Fig 6 *cannot occur in a minimal-ABC tree*.

**Proposition 20** ([27, Proposition 3.4]). *When* *s* = 0 *and* *t* > 3, *the configuration* *T* *depicted in* Fig 6 *cannot occur in a minimal-ABC tree*.

**Proposition 21**. *The configuration* *T* *depicted in* Fig 6 *cannot occur in a minimal-ABC tree, for the following cases*:

- *t* = 3 *and* *s* = 0, 4, 5;
- *t* = 4 *and* *s* = 2, 3, 4;
- *t* = 5 *and* *s* = 1, 2, 3;
- *t* = 6 *and* *s* = 1, 2;
- *t* = 7 *and* *s* = 1.

*proof.* Let *d*_*x*_ be the degree of vertex *x* in *T*.

**Fig 7:**
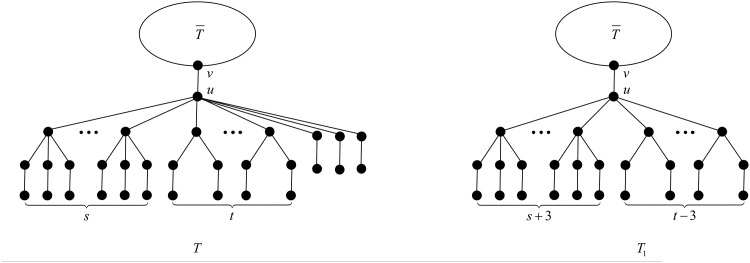
The transformation $T_{1}$ in the proof of Proposition 21.

First we apply the transformation $T_{1}$ illustrated in Fig 7.

After applying $T_{1}$, the degree of vertex *u* decreases by 3, while the degrees of three children of *u* in *T* belonging to a *B*_2_-branch increase from 3 to 4, and the rest of the vertices do not change their degrees. The change of the ABC index after applying $T_{1}$ is

$$\begin{array}{ccc} ABC\left(T_{1}\right)-ABC\left(T\right) & = & f\left(s+t+1,d_{v}\right)-f\left(s+t+4,d_{v}\right)\\ & & +(s+3)f(s+t+1,4)+(t-3)f(s+t+1,3)\\ & & -s\cdot f(s+t+4,4)-t\cdot f(s+t+4,3)\;. \end{array}$$

From Lemma 11, *f*(*s* + *t* + 1, *d*_*v*_) − *f*(*s* + *t* + 4, *d*_*v*_) increases in *d*_*v*_, and thus

$$\begin{array}{ccc} f\left(s+t+1,d_{v}\right)-f\left(s+t+4,d_{v}\right) & \leq & \lim_{d_{v}\to +\infty }\left(f\left(s+t+1,d_{v}\right)-f\left(s+t+4,d_{v}\right)\right)\\ & = & \sqrt{\frac{1}{s+t+1}}-\sqrt{\frac{1}{s+t+4}}\;. \end{array}$$

Now it follows that

$$\begin{array}{ccc} ABC\left(T_{1}\right)-ABC\left(T\right) & \leq & \sqrt{\frac{1}{s+t+1}}-\sqrt{\frac{1}{s+t+4}}\\ & & +(s+3)f(s+t+1,4)+(t-3)f(s+t+1,3)\\ & & -s\cdot f(s+t+4,4)-t\cdot f(s+t+4,3)\;. \end{array}$$ (6)

**Fig 8:**
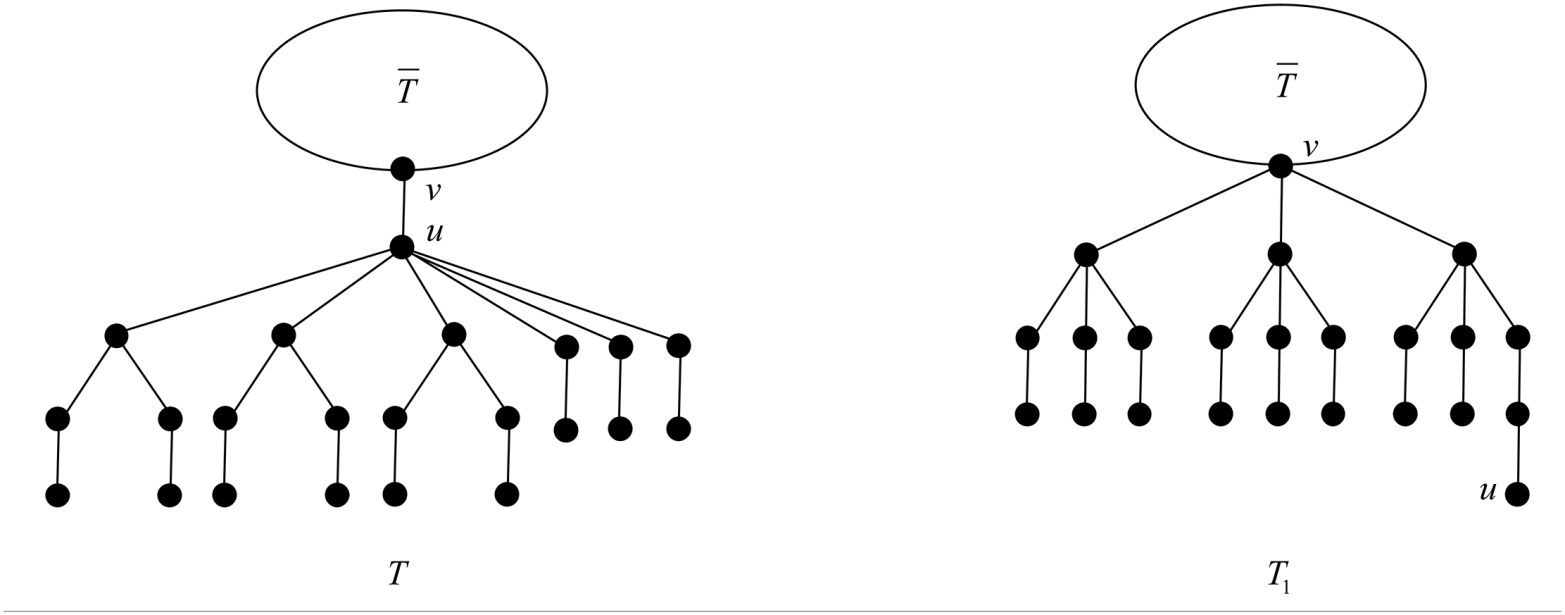
The transformation $T_{2}$ in the proof of Proposition 21.

By virtue of Mathematica, the right-hand side of (6) is negative, equivalently *ABC*(*T*_1_) < *ABC*(*T*), follows from direct calculation easily, except the case *t* = 3 and *s* = 0. In such case, we apply the transformation $T_{2}$ illustrated in Fig 8.

After applying $T_{2}$, the degree of vertex *v* increases by 2, the degrees of three children of *u* in *T* belonging to a *B*_2_-branch increase from 3 to 4, a pendent vertex in *T* belonging to a *B*_2_-branch increases its degree from 1 to 2, the degree of *u* decreases from 7 to 1, and the rest of the vertices do not change their degrees. The change of the ABC index after applying $T_{2}$ is

$$\begin{array}{ccc} ABC\left(T_{1}\right)-ABC\left(T\right) & = & \sum_{xv\in E(\bar{T})}\left(f\left(d_{v}+2,d_{x}\right)-f\left(d_{v},d_{x}\right)\right)\\ & & +f\left(2,1\right)-f\left(d_{v},7\right)+3\left(f\left(d_{v}+2,4\right)-f\left(7,3\right)\right)\;. \end{array}$$ (7)

Clearly, *f*(*d*_*v*_ + 2, *d*_*x*_) − *f*(*d*_*v*_, *d*_*x*_) < 0 for $xv\in E(\bar{T})$. So

$$\begin{array}{ccc} ABC\left(T_{1}\right)-ABC\left(T\right) & < & f(2,1-f\left(d_{v},7\right)+3\left(f\left(d_{v}+2,4\right)-f\left(7,3\right)\right)\\ & = & 3f\left(d_{v}+2,4\right)-f\left(d_{v},7\right)+f\left(2,1\right)-3f\left(7,3\right)\;. \end{array}$$

Note that *d*_*v*_ ≥ *d*_*u*_ = 7 from Proposition 2, and from Lemma 12, we know that 3*f*(*d*_*v*_ + 2, 4) − *f*(*d*_*v*_, 7) decreases in *d*_*v*_ ≥ 7. Therefore, for *d*_*v*_ ≥ 20, we get that

$$\begin{array}{ccc} ABC\left(T_{1}\right)-ABC\left(T\right) & < & 3f(20+2,4)-f(20,7)+f(2,1)-3f(7,3)<0\;. \end{array}$$

For the remaining cases that 7 ≤ *d*_*v*_ ≤ 19, let us be a bit more precisely in (7) for the term

$$\begin{array}{c} \sum_{xv\in E(\bar{T})}\left(f\left(d_{v}+2,d_{x}\right)-f\left(d_{v},d_{x}\right)\right)\;. \end{array}$$

Note that every neighbor of *v* in $\bar{T}$ has degree at least three from Proposition 2. By Lemma 10, *f*(*d*_*v*_ + 2, *d*_*x*_) − *f*(*d*_*v*_, *d*_*x*_) decreases in *d*_*x*_ ≥ 3, and thus

$$\begin{array}{c} \sum_{xv\in E(\bar{T})}\left(f\left(d_{v}+2,d_{x}\right)-f\left(d_{v},d_{x}\right)\right)\leq \left(d_{v}-1\right)(\left(f\left(d_{v}+2,3\right)-f\left(d_{v},3\right)\right)\;. \end{array}$$

Now together with (7), it follows that

$$\begin{array}{ccc} ABC\left(T_{1}\right)-ABC\left(T\right) & \leq & \left(d_{v}-1\right)(\left(f\left(d_{v}+2,3\right)-f\left(d_{v},3\right)\right)\\ & & +f\left(2,1\right)-f\left(d_{v},7\right)+3\left(f\left(d_{v}+2,4\right)-f\left(7,3\right)\right)\;. \end{array}$$ (8)

By virtue of Mathematica, the right-hand side of (8) is negative, equivalently *ABC*(*T*_1_) < *ABC*(*T*), for 7 ≤ *d*_*v*_ ≤ 19, follows from direct calculation easily.

Then the result follows.

We are now prepared to establish the main result of this section.

**Theorem 22**. *A minimal-ABC tree cannot contain three* *B*_1_-*branches*.

*proof.* Similarly to Theorem 18, let us suppose to the contrary that *T* is a minimal-ABC tree containing exactly three *B*_1_-branches. Observe that the three *B*_1_-branches are attached to the same vertex, say *u*, otherwise, there are at least two *T*_*k*_-branches, which is a contradiction to Proposition 8. Moreover, by Proposition 15, *u* is not the root vertex of *T*. Denote by *v* the parent of *u*.

First, by Proposition 3, *u* contains no *B*_*k*_-branch with *k* > 4. Next by Proposition 5, *u* contains no *B*_4_-branch, and by Propositions 4 and 7, *u* contains no $B_{1}^{*}$-branch, no matter *u* has *B*_3_-branches or *B*_2_-branches. Now we may deduce that the branches attached to *u* must be *B*_3_-, *B*_2_- or *B*_1_-branches, i.e., *T* is of the structure depicted in Fig 6.

Let us denote by *s* the number of *B*_3_-branches attached to *u*, and by *t* the number of *B*_2_-branches attached to *u*. Clearly, *s* + *t* ≥ 1, and *s* + *t* ≤ 8, from Proposition 19.

**Fig 9:**
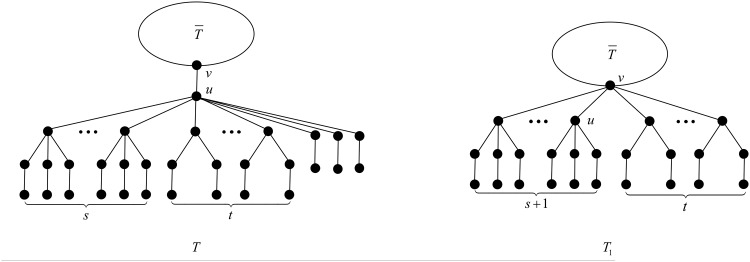
The transformation T in the proof of Theorem 22.

We apply the transformation T depicted in Fig 9. And let *d*_*x*_ be the degree of vertex *x* in *T*.

After applying T, the degree of vertex *v* increases by *s* + *t*, while the degree of vertex *u* decreases by *s* + *t*, and the rest of the vertices do not change their degrees. The change of the ABC index after applying T is

$$\begin{array}{ccc} ABC\left(T_{1}\right)-ABC\left(T\right) & = & \sum_{xv\in E(\bar{T})}\left(f\left(d_{v}+s+t,d_{x}\right)-f\left(d_{v},d_{x}\right)\right)\\ & & +s(f\left(d_{v}+s+t,4\right)-f\left(s+t+4,4\right))\\ & & +t(f\left(d_{v}+s+t,3\right)-f\left(s+t+4,3\right))\\ & & +f\left(d_{v}+s+t,4\right)-f\left(d_{v},s+t+4\right)\;. \end{array}$$ (9)

Clearly, *f*(*d*_*v*_ + *s* + *t*, *d*_*x*_) − *f*(*d*_*v*_, *d*_*x*_) < 0 for $xv\in E(\bar{T})$, and thus

$$\begin{array}{ccc} ABC\left(T_{1}\right)-ABC\left(T\right) & < & s(f\left(d_{v}+s+t,4\right)-f\left(s+t+4,4\right))\\ & & +t(f\left(d_{v}+s+t,3\right)-f\left(s+t+4,3\right))\\ & & +f\left(d_{v}+s+t,4\right)-f\left(d_{v},s+t+4\right)\;. \end{array}$$

On one hand, from Lemma 10, *f*(*d*_*v*_ + *s* + *t*, 4) − *f*(*d*_*v*_, *s* + *t* + 4) increases in *d*_*v*_, thus

$$\begin{array}{ccc} f\left(d_{v}+s+t,4\right)-f\left(d_{v},s+t+4\right) & \leq & \lim_{d_{v}\to +\infty }\left(f\left(d_{v}+s+t,4\right)-f\left(d_{v},s+t+4\right)\right)\\ & = & \sqrt{\frac{1}{4}}-\sqrt{\frac{1}{s+t+4}}\;. \end{array}$$

So it follows that

$$\begin{array}{ccc} ABC\left(T_{1}\right)-ABC\left(T\right) & < & s(f\left(d_{v}+s+t,4\right)-f\left(s+t+4,4\right))\\ & & +t(f\left(d_{v}+s+t,3\right)-f\left(s+t+4,3\right))\\ & & +\sqrt{\frac{1}{4}}-\sqrt{\frac{1}{s+t+4}}\;. \end{array}$$ (10)

On the other hand, note that *d*_*v*_ ≥ *d*_*u*_ = *s* + *t* + 4 from Proposition 2, and both *f*(*d*_*v*_ + *s* + *t*, 4) and *f*(*d*_*v*_ + *s* + *t*, 3) decrease in *d*_*v*_ ≥ *s* + *t* + 4, i.e., the right-hand side of (10) also decreases in *d*_*v*_ ≥ *s* + *t* + 4.

Besides the upper bound about *ABC*(*T*_1_) − *ABC*(*T*) as (10), by considering a bit precisely in (9) for the term

$$\begin{array}{c} \sum_{xv\in E(\bar{T})}\left(f\left(d_{v}+s+t,d_{x}\right)-f\left(d_{v},d_{x}\right)\right)\;, \end{array}$$

we may get a somewhat stricter upper bound about *ABC*(*T*_1_) − *ABC*(*T*). Note that, from Lemma 10, *f*(*d*_*v*_ + *s* + *t*, *d*_*x*_) − *f*(*d*_*v*_, *d*_*x*_) decreases in *d*_*x*_, and from Proposition 2, every neighbor of *v* in $\bar{T}$ has degree at least three, thus

$$\begin{array}{c} \sum_{xv\in E(\bar{T})}\left(f\left(d_{v}+s+t,d_{x}\right)-f\left(d_{v},d_{x}\right)\right)\leq \left(d_{v}-1\right)\left(f\left(d_{v}+s+t,3\right)-f\left(d_{v},3\right)\right)\;. \end{array}$$

Now together with (9), it follows that

$$\begin{array}{ccc} ABC\left(T_{1}\right)-ABC\left(T\right) & \leq & \left(d_{v}-1\right)\left(f\left(d_{v}+s+t,3\right)-f\left(d_{v},3\right)\right)\\ & & +s(f\left(d_{v}+s+t,4\right)-f\left(s+t+4,4\right))\\ & & +t(f\left(d_{v}+s+t,3\right)-f\left(s+t+4,3\right))\\ & & +f\left(d_{v}+s+t,4\right)-f\left(d_{v},s+t+4\right)\;. \end{array}$$ (11)

**Case 1.** *t* = 0.

In this case, note that 1 ≤ *s* ≤ 8, and *d*_*v*_ ≥ *s* + 4.

By direct calculation, we may deduce that the right-hand side of (10) is negative, equivalently *ABC*(*T*_1_) < *ABC*(*T*), holds for the following cases:

- *s* = 1 and *d*_*v*_ ≥ 12;
- *s* = 2 and *d*_*v*_ ≥ 14;
- *s* = 3 and *d*_*v*_ ≥ 16;
- *s* = 4 and *d*_*v*_ ≥ 18;
- *s* = 5 and *d*_*v*_ ≥ 21;
- *s* = 6 and *d*_*v*_ ≥ 23;
- *s* = 7 and *d*_*v*_ ≥ 26;
- *s* = 8 and *d*_*v*_ ≥ 26.

For the remaining cases as follows:

- *s* = 1 and 5 ≤ *d*_*v*_ ≤ 11;
- *s* = 2 and 6 ≤ *d*_*v*_ ≤ 13;
- *s* = 3 and 7 ≤ *d*_*v*_ ≤ 15;
- *s* = 4 and 8 ≤ *d*_*v*_ ≤ 17;
- *s* = 5 and 9 ≤ *d*_*v*_ ≤ 20;
- *s* = 6 and 10 ≤ *d*_*v*_ ≤ 22;
- *s* = 7 and 11 ≤ *d*_*v*_ ≤ 25;
- *s* = 8 and 12 ≤ *d*_*v*_ ≤ 25,

we would turn to use (11), and negative upper bounds, equivalently *ABC*(*T*_1_) < *ABC*(*T*), follow from direct calculation.

**Case 2.** *t* = 1.

In this case, note that 0 ≤ *s* ≤ 7, and *d*_*v*_ ≥ *s* + 5.

By direct calculation, we may deduce that the right-hand side of (10) is negative, equivalently *ABC*(*T*_1_) < *ABC*(*T*), holds for the following cases:

- *s* = 0 and *d*_*v*_ ≥ 124;
- *s* = 1 and *d*_*v*_ ≥ 23;
- *s* = 2 and *d*_*v*_ ≥ 22;
- *s* = 3 and *d*_*v*_ ≥ 22;
- *s* = 4 and *d*_*v*_ ≥ 25;
- *s* = 5 and *d*_*v*_ ≥ 28;
- *s* = 6 and *d*_*v*_ ≥ 30;
- *s* = 7 and *d*_*v*_ ≥ 33.

For the remaining cases as follows:

- *s* = 0 and 5 ≤ *d*_*v*_ ≤ 123;
- *s* = 1 and 6 ≤ *d*_*v*_ ≤ 22;
- *s* = 2 and 7 ≤ *d*_*v*_ ≤ 21;
- *s* = 3 and 8 ≤ *d*_*v*_ ≤ 21;
- *s* = 4 and 9 ≤ *d*_*v*_ ≤ 24;
- *s* = 5 and 10 ≤ *d*_*v*_ ≤ 27;
- *s* = 6 and 11 ≤ *d*_*v*_ ≤ 29;
- *s* = 7 and 12 ≤ *d*_*v*_ ≤ 32,

we would turn to use (11), and negative upper bounds, equivalently *ABC*(*T*_1_) < *ABC*(*T*), follow from direct calculation easily.

**Case 3.** *t* = 2.

In this case, note that 0 ≤ *s* ≤ 6, and *d*_*v*_ ≥ *s* + 6.

By direct calculation, we may deduce that the right-hand side of (10) is negative, equivalently *ABC*(*T*_1_) < *ABC*(*T*), holds for the following cases:

- *s* = 0 and *d*_*v*_ ≥ 751;
- *s* = 1 and *d*_*v*_ ≥ 41;
- *s* = 2 and *d*_*v*_ ≥ 34;
- *s* = 3 and *d*_*v*_ ≥ 33;
- *s* = 4 and *d*_*v*_ ≥ 35;
- *s* = 5 and *d*_*v*_ ≥ 37;
- *s* = 6 and *d*_*v*_ ≥ 39.

For the remaining cases as follows:

- *s* = 0 and 6 ≤ *d*_*v*_ ≤ 750;
- *s* = 1 and 7 ≤ *d*_*v*_ ≤ 40;
- *s* = 2 and 8 ≤ *d*_*v*_ ≤ 33;
- *s* = 3 and 9 ≤ *d*_*v*_ ≤ 32;
- *s* = 4 and 10 ≤ *d*_*v*_ ≤ 34;
- *s* = 5 and 11 ≤ *d*_*v*_ ≤ 36;
- *s* = 6 and 12 ≤ *d*_*v*_ ≤ 38,

we would turn to use (11), and negative upper bounds, equivalently *ABC*(*T*_1_) < *ABC*(*T*), follow from direct calculation easily.

**Case 4.** *t* = 3.

In this case, note that 0 ≤ *s* ≤ 5, and *d*_*v*_ ≥ *s* + 7.

On one hand, the contradiction for the cases *s* = 0, 4, 5 may be deduced from Proposition 21.

On the other hand, by direct calculation, we may deduce that the right-hand side of (10) is negative, equivalently *ABC*(*T*_1_) < *ABC*(*T*), holds for the following cases:

- *s* = 1 and *d*_*v*_ ≥ 74;
- *s* = 2 and *d*_*v*_ ≥ 52;
- *s* = 3 and *d*_*v*_ ≥ 48.

For the remaining cases as follows:

- *s* = 1 and 8 ≤ *d*_*v*_ ≤ 73;
- *s* = 2 and 9 ≤ *d*_*v*_ ≤ 51;
- *s* = 3 and 10 ≤ *d*_*v*_ ≤ 47,

we would turn to use (11), and negative upper bounds, equivalently *ABC*(*T*_1_) < *ABC*(*T*), follow from direct calculation easily.

**Case 5.** *t* = 4.

In this case, note that 0 ≤ *s* ≤ 4, and *d*_*v*_ ≥ *s* + 8.

The contradiction for the cases *s* = 0 and *s* = 2, 3, 4 may be, respectively, deduced from Propositions 20 and 21.

Besides that, by direct calculation, we may deduce that the right-hand side of (10) is negative, equivalently *ABC*(*T*_1_) < *ABC*(*T*), for *s* = 1 and *d*_*v*_ ≥ 145. For the remaining cases *s* = 1 and 9 ≤ *d*_*v*_ ≤ 144, we would turn to use (11), and a negative upper bound, equivalently *ABC*(*T*_1_) < *ABC*(*T*), follows from direct calculation easily.

**Case 6.** *t* = 5.

In this case, note that *s* = 0, 1, 2, 3. The contradiction for the cases *s* = 0 and *s* = 1, 2, 3 may be, respectively, deduced from Propositions 20 and 21.

**Case 7.** *t* = 6.

In this case, note that *s* = 0, 1, 2. The contradiction for the cases *s* = 0 and *s* = 1, 2 may be, respectively, deduced from Propositions 20 and 21.

**Case 8.** *t* = 7.

In this case, note that *s* = 0, 1. The contradiction for the cases *s* = 0 and *s* = 1 may be, respectively, deduced from Propositions 20 and 21.

**Case 9.** *t* = 8.

In this case, note that *s* = 0. The contradiction may be deduced from Proposition 20 directly.

Combining the above cases, the result follows.

### The existence of two *B*_1_-branches

This last section is devoted to the analysis of the existence of two *B*_1_-branches in a minimal-ABC tree. The first two propositions are known results establishing forbidden configurations in such cases.

**Fig 10:**
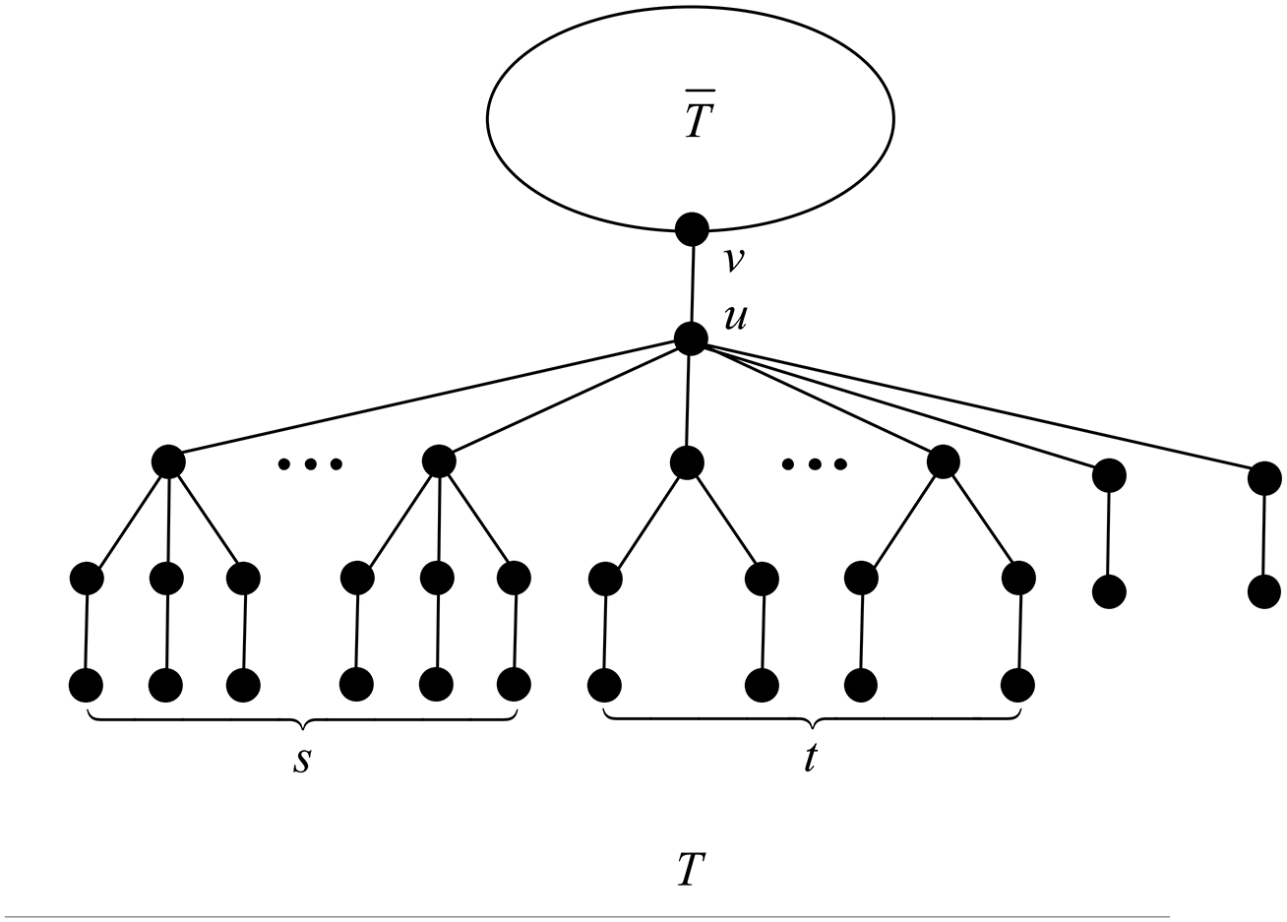
The tree *T* in Propositions 23, 24 and 25, and Theorem 26.

**Proposition 23** ([27, Proposition 3.2]). *When* *s* + *t* > 10, *the configuration* *T* *depicted in* Fig 10 *cannot occur in a minimal-ABC tree*.

**Proposition 24** ([27, Proposition 3.4]). *When* *s* = 0 *and* *t* > 4, *the configuration* *T* *depicted in* Fig 10 *cannot occur in a minimal-ABC tree*.

We next list several cases more where the configuration depicted in Fig 10 is not possible in a minimal-ABC tree.

**Proposition 25**. *The configuration* *T* *depicted in* Fig 10 *cannot occur in a minimal-ABC tree, for the following cases*:

- *t* = 2 *and* *s* = 0;
- *t* = 3 *and* *s* = 1, 2;
- *t* = 4 *and* *s* = 0, 1, 2, 3, 4, 5, 6;
- *t* = 5 *and* *s* = 1, 2, 3, 4, 5;
- *t* = 6 *and* *s* = 1, 2, 3, 4;
- *t* = 7 *and* *s* = 1, 2, 3;
- *t* = 8 *and* *s* = 1, 2;
- *t* = 9 *and* *s* = 1.

**Fig 11:**
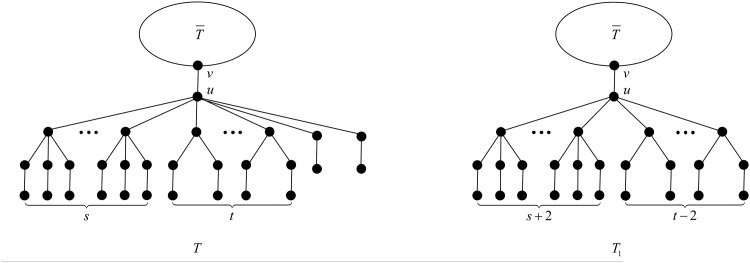
The transformation $T_{1}$ in the proof of Proposition 25.

*proof.* First we apply the transformation $T_{1}$ illustrated in Fig 11. Let *d*_*x*_ be the degree of vertex *x* in *T*.

After applying $T_{1}$, the degree of vertex *u* decreases by 2, while the degrees of two children of *u* in *T* belonging to a *B*_2_-branch increase from 3 to 4. The rest of the vertices do not change their degrees. The change of the ABC index after applying $T_{1}$ is

$$\begin{array}{ccc} ABC\left(T_{1}\right)-ABC\left(T\right) & = & f\left(s+t+1,d_{v}\right)-f\left(s+t+3,d_{v}\right)\\ & & +(s+2)f(s+t+1,4)+(t-2)f(s+t+1,3)\\ & & -s\cdot f(s+t+3,4)-t\cdot f(s+t+3,3)\;. \end{array}$$

From Lemma 11, *f*(*s* + *t* + 1, *d*_*v*_) − *f*(*s* + *t* + 3, *d*_*v*_) increases in *d*_*v*_, and thus

$$\begin{array}{ccc} f\left(s+t+1,d_{v}\right)-f\left(s+t+3,d_{v}\right) & \leq & \lim_{d_{v}\to +\infty }\left(f\left(s+t+1,d_{v}\right)-f\left(s+t+3,d_{v}\right)\right)\\ & = & \sqrt{\frac{1}{s+t+1}}-\sqrt{\frac{1}{s+t+3}}\;. \end{array}$$

Now it follows that

$$\begin{array}{ccc} ABC\left(T_{1}\right)-ABC\left(T\right) & \leq & \sqrt{\frac{1}{s+t+1}}-\sqrt{\frac{1}{s+t+3}}\\ & & +(s+2)f(s+t+1,4)+(t-2)f(s+t+1,3)\\ & & -s\cdot f(s+t+3,4)-t\cdot f(s+t+3,3)\;. \end{array}$$ (12)

The right-hand side of (12) is negative, equivalently *ABC*(*T*_1_) < *ABC*(*T*), holds for the following cases:

- *t* = 4 and *s* = 3, 4, 5, 6;
- *t* = 5 and *s* = 2, 3, 4, 5;
- *t* = 6 and *s* = 1, 2, 3, 4;
- *t* = 7 and *s* = 1, 2, 3;
- *t* = 8 and *s* = 1, 2;
- *t* = 9 and *s* = 1.

**Fig 12:**
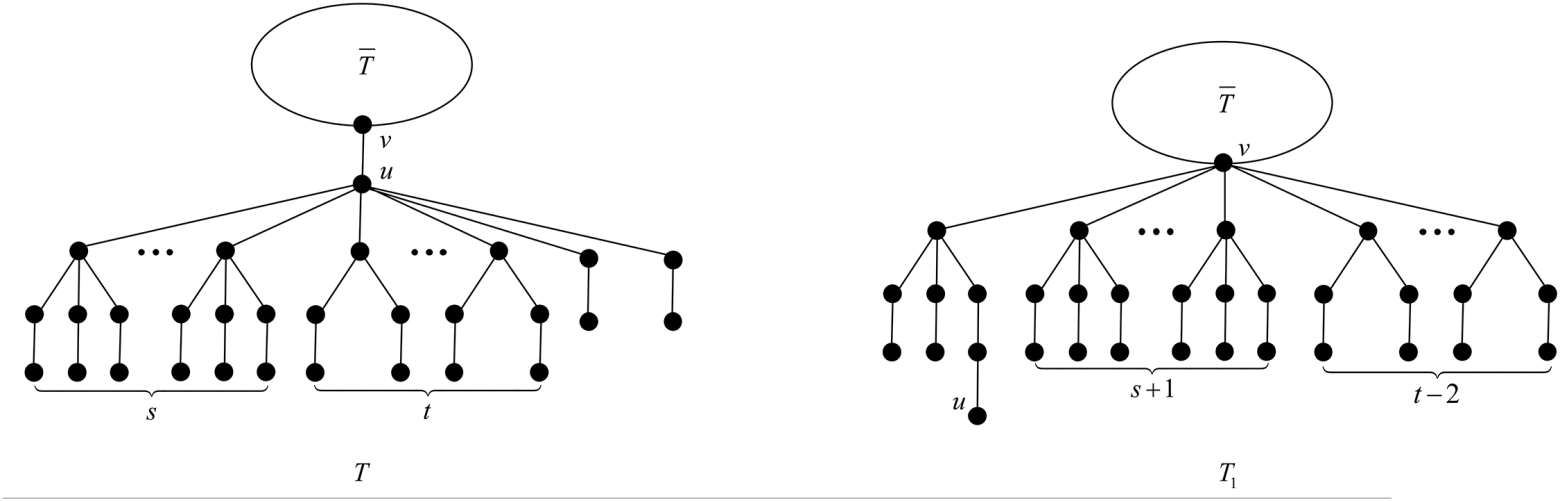
The transformation $T_{2}$ in the proof of Proposition 25.

Next for the following cases:

- *t* = 2 and *s* = 0;
- *t* = 3 and *s* = 1, 2;
- *t* = 4 and *s* = 1, 2,

we apply the transformation $T_{2}$ illustrated in Fig 12.

After applying $T_{2}$, the degree of vertex *v* increases by *s* + *t* − 1, the degrees of two children of *u* in *T* belonging to a *B*_2_-branch increase from 3 to 4, a pendent vertex in *T* belonging to a *B*_3_-branch increases its degree from 1 to 2, the degree of *u* decreases from *s* + *t* + 3 to 1, and the rest of the vertices do not change their degrees. The change of the ABC index after applying $T_{2}$ is

$$\begin{array}{ccc} ABC\left(T_{1}\right)-ABC\left(T\right) & = & \sum_{xv\in E(\bar{T})}\left(f\left(d_{v}+s+t-1,d_{x}\right)-f\left(d_{v},d_{x}\right)\right)\\ & & +s(f\left(d_{v}+s+t-1,4\right)-f\left(s+t+3,4\right))\\ & & +t(f\left(d_{v}+s+t-1,3\right)-f\left(s+t+3,3\right))\\ & & +2(f\left(d_{v}+s+t-1,4\right)-f\left(d_{v}+s+t-1,3\right))\\ & & +f\left(1,2\right)-f\left(d_{v},s+t+3\right)\;. \end{array}$$ (13)

Clearly, *f*(*d*_*v*_ + *s* + *t* − 1, *d*_*x*_) − *f*(*d*_*v*_, *d*_*x*_)≤0, for $xv\in E(\bar{T})$. So

$$\begin{array}{ccc} ABC\left(T_{1}\right)-ABC\left(T\right) & \leq & s(f\left(d_{v}+s+t-1,4\right)-f\left(s+t+3,4\right))\\ & & +t(f\left(d_{v}+s+t-1,3\right)-f\left(s+t+3,3\right))\\ & & +2(f\left(d_{v}+s+t-1,4\right)-f\left(d_{v}+s+t-1,3\right))\\ & & +f\left(1,2\right)-f\left(d_{v},s+t+3\right)\\ & = & \left(s+t\right)f\left(d_{v}+s+t-1,3\right)-f\left(d_{v},s+t+3\right)\\ & & +\left(s+2\right)(f\left(d_{v}+s+t-1,4\right)-f\left(d_{v}+s+t-1,3\right))\\ & & -s\cdot f(s+t+3,4)-t\cdot f(s+t+3,3)+f(1,2)\;. \end{array}$$ (14)

Note that *d*_*v*_ ≥ *d*_*u*_ = *s* + *t* + 3 from Proposition 2, and from Lemma 13, we know that (*s* + *t*)*f*(*d*_*v*_ + *s* + *t* − 1, 3) − *f*(*d*_*v*_, *s* + *t* + 3) increases in *d*_*v*_ ≥ 5 when *t* = 2 and *s* = 0, and decreases in

- *d*_*v*_ ≥ 19 when *t* = 3 and *s* = 1;
- *d*_*v*_ ≥ 17 when *t* = 3 and *s* = 2, or *t* = 4 and *s* = 1;
- *d*_*v*_ ≥ 16 when *t* = 4 and *s* = 2.

On the other hand, from Lemma 11, *f*(*d*_*v*_ + *s* + *t* − 1, 4) − *f*(*d*_*v*_ + *s* + *t* − 1, 3) also decreases in *d*_*v*_ ≥ *s* + *t* + 3.

So if *t* = 2 and *s* = 0, and *d*_*v*_ ≥ 83, then by (14),

$$\begin{array}{ccc} ABC\left(T_{1}\right)-ABC\left(T\right) & \leq & 2f\left(d_{v}+1,3\right)-f\left(d_{v},5\right)\\ & & +2(f\left(d_{v}+1,4\right)-f\left(d_{v}+1,3\right))\\ & & -2f(5,3)+f(1,2)\\ & \leq & \lim_{d_{v}\to +\infty }\left(2f\left(d_{v}+1,3\right)-f\left(d_{v},5\right)\right)\\ & & +2(f(83+1,4)-f(83+1,3))\\ & & -2f(5,3)+f(1,2)\\ & < & 0\;. \end{array}$$

Otherwise, the right-hand of (14) decreases in the following cases:

- *d*_*v*_ ≥ 19 when *t* = 3 and *s* = 1;
- *d*_*v*_ ≥ 17 when *t* = 3 and *s* = 2 or *t* = 4 and *s* = 1;
- *d*_*v*_ ≥ 16 when *t* = 4 and *s* = 2.

Besides the upper bound about *ABC*(*T*_1_) − *ABC*(*T*) as (14), by considering in particular in (13) the term

$$\begin{array}{c} \sum_{xv\in E(\bar{T})}\left(f\left(d_{v}+s+t-1,d_{x}\right)-f\left(d_{v},d_{x}\right)\right)\;, \end{array}$$

we may get a somewhat stricter upper bound about *ABC*(*T*_1_) − *ABC*(*T*). Note that, from Lemma 10, *f*(*d*_*v*_ + *s* + *t* − 1, *d*_*x*_) − *f*(*d*_*v*_, *d*_*x*_) decreases in *d*_*x*_, and from Proposition 2, every neighbor of *v* in $\bar{T}$ has degree at least three, thus

$$\begin{array}{c} \sum_{xv\in E(\bar{T})}\left(f\left(d_{v}+s+t-1,d_{x}\right)-f\left(d_{v},d_{x}\right)\right)\leq \left(d_{v}-1\right)\left(f\left(d_{v}+s+t-1,3\right)-f\left(d_{v},3\right)\right)\;. \end{array}$$

Now together with (13), it follows that

$$\begin{array}{ccc} ABC\left(T_{1}\right)-ABC\left(T\right) & \leq & \left(d_{v}-1\right)\left(f\left(d_{v}+s+t-1,3\right)-f\left(d_{v},3\right)\right)\\ & & +s(f\left(d_{v}+s+t-1,4\right)-f\left(s+t+3,4\right))\\ & & +t(f\left(d_{v}+s+t-1,3\right)-f\left(s+t+3,3\right))\\ & & +2(f\left(d_{v}+s+t-1,4\right)-f\left(d_{v}+s+t-1,3\right))\\ & & +f\left(1,2\right)-f\left(d_{v},s+t+3\right)\;. \end{array}$$ (15)

By direct calculation, we may deduce that the right-hand side of (14) is negative, equivalently *ABC*(*T*_1_) < *ABC*(*T*), holds for the following cases:

- *t* = 3, *s* = 1, and *d*_*v*_ ≥ 64;
- *t* = 3, *s* = 2, and *d*_*v*_ ≥ 44;
- *t* = 4, *s* = 1, and *d*_*v*_ ≥ 4015;
- *t* = 4, *s* = 2, and *d*_*v*_ ≥ 116.

For the remaining cases as follows:

- *t* = 2, *s* = 0, and 5 ≤ *d*_*v*_ ≤ 82;
- *t* = 3, *s* = 1, and 7 ≤ *d*_*v*_ ≤ 63;
- *t* = 3, *s* = 2, and 8 ≤ *d*_*v*_ ≤ 43;
- *t* = 4, *s* = 1, and 8 ≤ *d*_*v*_ ≤ 4014;
- *t* = 4, *s* = 2, and 9 ≤ *d*_*v*_ ≤ 115,

we would turn to use (15), and negative upper bounds, equivalently *ABC*(*T*_1_) < *ABC*(*T*), follow from direct calculation easily.

At this point, there are still two remaining cases: *t* = 4, *s* = 0, and *t* = 5, *s* = 1.

**Fig 13:**
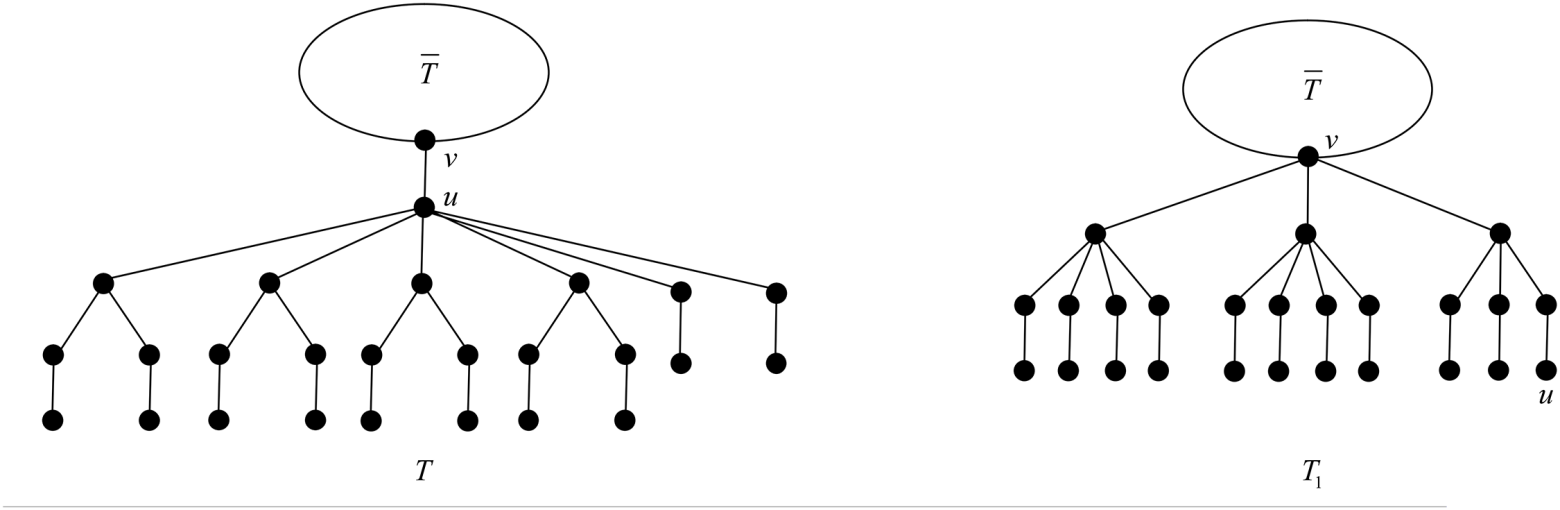
The transformation $T_{3}$ in the proof of Proposition 25.

For the case *t* = 4 and *s* = 0, we apply the transformation $T_{3}$ illustrated in Fig 13.

After applying $T_{3}$, the degree of vertex *v* increases by 2, the degrees of two children of *u* in *T* belonging to a *B*_2_-branch increase from 3 to 5, one child of *u* in *T* belonging to another *B*_2_-branch increases its degree from 3 to 4, the remaining child of *u* in *T* belonging to a *B*_2_-branch decreases its degree from 3 to 2, the degree of *u* decreases from 7 to 1, and the rest of the vertices do not change their degrees. The change of the ABC index after applying $T_{3}$ is

$$\begin{array}{ccc} ABC\left(T_{1}\right)-ABC\left(T\right) & = & \sum_{xv\in E(\bar{T})}\left(f\left(d_{v}+2,d_{x}\right)-f\left(d_{v},d_{x}\right)\right)\\ & & +2\left(f\left(d_{v}+2,5\right)-f\left(7,3\right)\right)+f\left(d_{v}+2,4\right)-f\left(d_{v},7\right)\\ & & +2(f(1,2)-f(7,3))\;. \end{array}$$ (16)

Clearly,

$$\begin{array}{c} f\left(d_{v}+2,d_{x}\right)-f\left(d_{v},d_{x}\right)<0\;, \end{array}$$

for $xv\in E(\bar{T})$. So

$$\begin{array}{ccc} ABC\left(T_{1}\right)-ABC\left(T\right) & < & 2\left(f\left(d_{v}+2,5\right)-f\left(7,3\right)\right)+f\left(d_{v}+2,4\right)-f\left(d_{v},7\right)\\ & & +2(f(1,2)-f(7,3))\\ & = & 2\left(f\left(d_{v}+2,5\right)-f\left(d_{v}+2,4\right)\right)+3f\left(d_{v}+2,4\right)-f\left(d_{v},7\right)\\ & & -4f(7,3)+2f(1,2)\;. \end{array}$$ (17)

Note that *d*_*v*_ ≥ *d*_*u*_ = 7 from Proposition 2, and by Lemma 11, *f*(*d*_*v*_ + 2, 5) − *f*(*d*_*v*_ + 2, 4) decreases in *d*_*v*_ ≥ 7. On the other hand, by Lemma 12, 3*f*(*d*_*v*_ + 2, 4) − *f*(*d*_*v*_, 7) decreases in *d*_*v*_ ≥ 7. So the right-hand side of (17) also decreases in *d*_*v*_ ≥ 7.

Besides the upper bound about *ABC*(*T*_1_) − *ABC*(*T*) as (17), by considering in (16) the term

$$\begin{array}{c} \sum_{xv\in E(\bar{T})}\left(f\left(d_{v}+2,d_{x}\right)-f\left(d_{v},d_{x}\right)\right)\;, \end{array}$$

we may get a somewhat stricter upper bound about *ABC*(*T*_1_) − *ABC*(*T*). Note that, from Lemma 10, *f*(*d*_*v*_ + 2, *d*_*x*_) − *f*(*d*_*v*_, *d*_*x*_) decreases in *d*_*x*_, and from Proposition 2, every neighbor of *v* in $\bar{T}$ has degree at least three, thus

$$\begin{array}{c} \sum_{xv\in E(\bar{T})}\left(f\left(d_{v}+2,d_{x}\right)-f\left(d_{v},d_{x}\right)\right)\leq \left(d_{v}-1\right)\left(f\left(d_{v}+2,3\right)-f\left(d_{v},3\right)\right)\;. \end{array}$$

Now together with (16), it follows that

$$\begin{array}{ccc} ABC\left(T_{1}\right)-ABC\left(T\right) & \leq & \left(d_{v}-1\right)\left(f\left(d_{v}+2,3\right)-f\left(d_{v},3\right)\right)\\ & & +2\left(f\left(d_{v}+2,5\right)-f\left(7,3\right)\right)+f\left(d_{v}+2,4\right)-f\left(d_{v},7\right)\\ & & +2(f(1,2)-f(7,3))\;. \end{array}$$ (18)

For *d*_*v*_ ≥ 20, by (17), we have

$$\begin{array}{ccc} ABC\left(T_{1}\right)-ABC\left(T\right) & < & 2(f(20+2,5)-f(7,3))+f(20+2,4)-f(20,7)\\ & & +2(f(1,2)-f(7,3))\\ & < & 0\;, \end{array}$$

i.e., *ABC*(*T*_1_) < *ABC*(*T*). For the remaining cases 7 ≤ *d*_*v*_ ≤ 19, we would turn to use (18), and a negative upper bound, equivalently *ABC*(*T*_1_) < *ABC*(*T*), follows from direct calculation straightforwardly.

**Fig 14:**
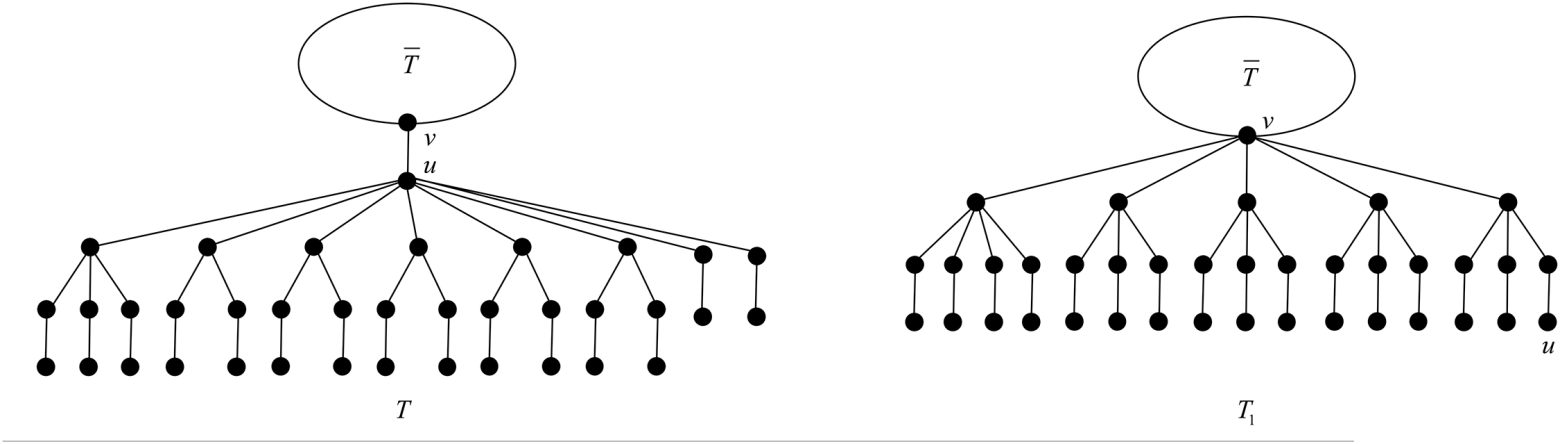
The transformation $T_{4}$ in the proof of Proposition 25.

As to the last case *t* = 5 and *s* = 1, we apply the transformation $T_{4}$ illustrated in Fig 14.

After applying $T_{4}$, the degree of vertex *v* increases by 4, three children of *u* in *T* belonging to a *B*_2_-branch increase its degrees from 3 to 4, the degree of one child of *u* in *T* belonging to another *B*_2_-branch increases from 3 to 5, the remaining child of *u* in *T* belonging to a *B*_2_-branch decreases its degree from 3 to 2, the degree of *u* decreases from 9 to 1, and the rest of the vertices do not change their degrees. The change of the ABC index after applying $T_{4}$ is

$$\begin{array}{ccc} ABC\left(T_{1}\right)-ABC\left(T\right) & = & \sum_{xv\in E(\bar{T})}\left(f\left(d_{v}+4,d_{x}\right)-f\left(d_{v},d_{x}\right)\right)\\ & & +4\left(f\left(d_{v}+4,4\right)-f\left(9,3\right)\right)+f\left(d_{v}+4,5\right)-f\left(d_{v},9\right)\\ & & +2f(1,2)-f(9,4)-f(9,3)\;. \end{array}$$ (19)

Clearly, *f*(*d*_*v*_ + 4, *d*_*x*_) − *f*(*d*_*v*_, *d*_*x*_) < 0 for $xv\in E(\bar{T})$. So

$$\begin{array}{ccc} ABC\left(T_{1}\right)-ABC\left(T\right) & < & 4\left(f\left(d_{v}+4,4\right)-f\left(9,3\right)\right)+f\left(d_{v}+4,5\right)-f\left(d_{v},9\right)\\ & & +2f(1,2)-f(9,4)-f(9,3)\\ & = & f\left(d_{v}+4,5\right)-f\left(d_{v}+4,4\right)+5f\left(d_{v}+4,4\right)-f\left(d_{v},9\right)\\ & & -5f(9,3)-f(9,4)+2f(1,2)\;. \end{array}$$ (20)

Note that *d*_*v*_ ≥ *d*_*u*_ = 9 from Proposition 2, and by Lemma 11, *f*(*d*_*v*_ + 4, 5) − *f*(*d*_*v*_ + 4, 4) decreases in *d*_*v*_ ≥ 9. On the other hand, by Lemma 12, 5*f*(*d*_*v*_ + 4, 4) − *f*(*d*_*v*_, 9) decreases in *d*_*v*_ ≥ 9. So the right-hand side of (20) also decreases in *d*_*v*_ ≥ 9.

Besides the upper bound about *ABC*(*T*_1_) − *ABC*(*T*) as (20), by considering in (19) the term

$$\begin{array}{c} \sum_{xv\in E(\bar{T})}\left(f\left(d_{v}+4,d_{x}\right)-f\left(d_{v},d_{x}\right)\right)\;, \end{array}$$

we may get a somewhat stricter upper bound about *ABC*(*T*_1_) − *ABC*(*T*). Note that, from Lemma 10, *f*(*d*_*v*_ + 4, *d*_*x*_) − *f*(*d*_*v*_, *d*_*x*_) decreases in *d*_*x*_, and from Proposition 2, every neighbor of *v* in $\bar{T}$ has degree at least three, thus

$$\begin{array}{c} \sum_{xv\in E(\bar{T})}\left(f\left(d_{v}+4,d_{x}\right)-f\left(d_{v},d_{x}\right)\right)\leq \left(d_{v}-1\right)\left(f\left(d_{v}+4,3\right)-f\left(d_{v},3\right)\right)\;. \end{array}$$

Now together with (19), it follows that

$$\begin{array}{ccc} ABC\left(T_{1}\right)-ABC\left(T\right) & \leq & \left(d_{v}-1\right)\left(f\left(d_{v}+4,3\right)-f\left(d_{v},3\right)\right)\\ & & +4\left(f\left(d_{v}+4,4\right)-f\left(9,3\right)\right)+f\left(d_{v}+4,5\right)-f\left(d_{v},9\right)\\ & & +2f(1,2)-f(9,4)-f(9,3)\;. \end{array}$$ (21)

For *d*_*v*_ ≥ 15, by (20), we have

$$\begin{array}{ccc} ABC\left(T_{1}\right)-ABC\left(T\right) & < & f(15+4,5)-f(15+4,4)+5f(15+4,4)-f(15,9)\\ & & -5f(9,3)-f(9,4)+2f(1,2)\\ & < & 0\;, \end{array}$$

i.e., *ABC*(*T*_1_) < *ABC*(*T*). For the remaining cases 9 ≤ *d*_*v*_ ≤ 14, we would turn to use (21), and a negative upper bound, equivalently *ABC*(*T*_1_) < *ABC*(*T*), follows from direct calculation easily.

Combining the above arguments, the result follows.

Our main result is stated next. As we will see, the configuration depicted in Fig 10 is very important since, minimal-ABC trees may contain two *B*_1_-branches only in two very particular configurations.

**Theorem 26**. *A minimal-ABC tree cannot contain two* *B*_1_-*branches, unless the two* *B*_1_-*branches belong to the configuration depicted in* Fig 10 *with* *s* = 0, *t* = 1, *or* *s* = 0, *t* = 3.

*proof.* Suppose to the contrary that *T* is a minimal-ABC tree containing exactly two *B*_1_-branches. Observe that the two *B*_1_-branches are attached to the same vertex, say *u*, otherwise, there are at least two *T*_*k*_-branches, which is a contradiction to Proposition 8. Moreover, by Proposition 15, *u* is not the root vertex of *T*. Denote by *v* the parent of *u*.

First, by Proposition 3, *u* contains no *B*_*k*_-branch with *k* > 4. Next by Proposition 5, *u* contains no *B*_4_-branch, and by Propositions 4 and 7, *u* contains no $B_{1}^{*}$-branch, no matter *u* has *B*_3_-branches or *B*_2_-branches. Now we may deduce that the branches attached to *u* must be *B*_3_-, *B*_2_- or *B*_1_-branches, i.e., *T* is of the structure depicted in Fig 10.

Set *s* and *t* for the numbers of *B*_3_- and *B*_2_-branches attached to *u*, respectively. Clearly, *s* + *t* ≥ 1, and *s* + *t* ≤ 10 from Proposition 23.

**Fig 15:**
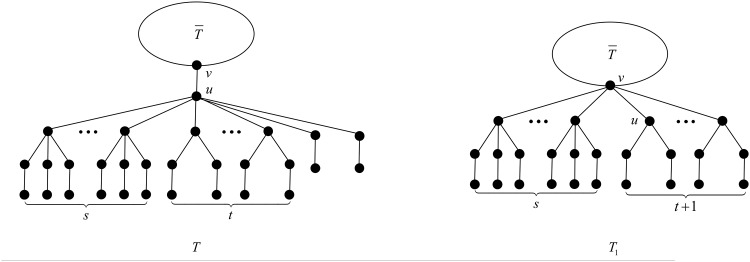
The transformation T in the proof of Theorem 26.

We apply the transformation T depicted in Fig 15. And let *d*_*x*_ be the degree of vertex *x* in *T*.

After applying T, the degree of vertex *v* increases by *s* + *t*, while the degree of vertex *u* decreases by *s* + *t*, and the rest of the vertices do not change their degrees. The change of the ABC index after applying T is

$$\begin{array}{ccc} ABC\left(T_{1}\right)-ABC\left(T\right) & = & \sum_{xv\in E(\bar{T})}\left(f\left(d_{v}+s+t,d_{x}\right)-f\left(d_{v},d_{x}\right)\right)\\ & & +s(f\left(d_{v}+s+t,4\right)-f\left(s+t+3,4\right))\\ & & +t(f\left(d_{v}+s+t,3\right)-f\left(s+t+3,3\right))\\ & & +f\left(d_{v}+s+t,3\right)-f\left(d_{v},s+t+3\right)\;. \end{array}$$ (22)

Clearly, *f*(*d*_*v*_ + *s* + *t*, *d*_*x*_) − *f*(*d*_*v*_, *d*_*x*_) < 0 for $xv\in E(\bar{T})$, and thus

$$\begin{array}{ccc} ABC\left(T_{1}\right)-ABC\left(T\right) & < & s(f\left(d_{v}+s+t,4\right)-f\left(s+t+3,4\right))\\ & & +t(f\left(d_{v}+s+t,3\right)-f\left(s+t+3,3\right))\\ & & +f\left(d_{v}+s+t,3\right)-f\left(d_{v},s+t+3\right)\;. \end{array}$$

On one hand, from Lemma 10, *f*(*d*_*v*_ + *s* + *t*, 3) − *f*(*d*_*v*_, *s* + *t* + 3) increases in *d*_*v*_, thus

$$\begin{array}{ccc} f\left(d_{v}+s+t,3\right)-f\left(d_{v},s+t+3\right) & \leq & \lim_{d_{v}\to +\infty }\left(f\left(d_{v}+s+t,3\right)-f\left(d_{v},s+t+3\right)\right)\\ & = & \sqrt{\frac{1}{3}}-\sqrt{\frac{1}{s+t+3}}\;. \end{array}$$

So it follows that

$$\begin{array}{ccc} ABC\left(T_{1}\right)-ABC\left(T\right) & < & s(f\left(d_{v}+s+t,4\right)-f\left(s+t+3,4\right))\\ & & +t(f\left(d_{v}+s+t,3\right)-f\left(s+t+3,3\right))\\ & & +\sqrt{\frac{1}{3}}-\sqrt{\frac{1}{s+t+3}}\;. \end{array}$$ (23)

Furthermore, note that *d*_*v*_ ≥ *d*_*u*_ = *s* + *t* + 3 from Proposition 2, and both *f*(*d*_*v*_ + *s* + *t*, 4) and *f*(*d*_*v*_ + *s* + *t*, 3) decrease in *d*_*v*_ ≥ *s* + *t* + 3, i.e., the right-hand side of (23) also decreases in *d*_*v*_ ≥ *s* + *t* + 3.

Besides the upper bound about *ABC*(*T*_1_) − *ABC*(*T*) as (23), by considering a bit precisely in (22) for the term

$$\begin{array}{c} \sum_{xv\in E(\bar{T})}\left(f\left(d_{v}+s+t,d_{x}\right)-f\left(d_{v},d_{x}\right)\right)\;, \end{array}$$

we may get a somewhat stricter upper bound about *ABC*(*T*_1_) − *ABC*(*T*). Note that, from Lemma 10, *f*(*d*_*v*_ + *s* + *t*, *d*_*x*_) − *f*(*d*_*v*_, *d*_*x*_) decreases in *d*_*x*_, and from Proposition 2, every neighbor of *v* in $\bar{T}$ has degree at least three, thus

$$\begin{array}{c} \sum_{xv\in E(\bar{T})}\left(f\left(d_{v}+s+t,d_{x}\right)-f\left(d_{v},d_{x}\right)\right)\leq \left(d_{v}-1\right)\left(f\left(d_{v}+s+t,3\right)-f\left(d_{v},3\right)\right)\;. \end{array}$$

Now together with (22), it follows that

$$\begin{array}{ccc} ABC\left(T_{1}\right)-ABC\left(T\right) & \leq & \left(d_{v}-1\right)\left(f\left(d_{v}+s+t,3\right)-f\left(d_{v},3\right)\right)\\ & & +s(f\left(d_{v}+s+t,4\right)-f\left(s+t+3,4\right))\\ & & +t(f\left(d_{v}+s+t,3\right)-f\left(s+t+3,3\right))\\ & & +f\left(d_{v}+s+t,3\right)-f\left(d_{v},s+t+3\right)\;. \end{array}$$ (24)

**Case 1.** *t* = 0.

In this case, note that 1 ≤ *s* ≤ 10, and *d*_*v*_ ≥ *s* + 3 from Proposition 2.

By direct calculation, we may deduce that the right-hand side of (23) is negative, equivalently *ABC*(*T*_1_) < *ABC*(*T*), holds for the following cases:

- *s* = 1 and *d*_*v*_ ≥ 13;
- *s* = 2 and *d*_*v*_ ≥ 17;
- *s* = 3 and *d*_*v*_ ≥ 21;
- *s* = 4 and *d*_*v*_ ≥ 25;
- *s* = 5 and *d*_*v*_ ≥ 30;
- *s* = 6 and *d*_*v*_ ≥ 35;
- *s* = 7 and *d*_*v*_ ≥ 41;
- *s* = 8 and *d*_*v*_ ≥ 47;
- *s* = 9 and *d*_*v*_ ≥ 54;
- *s* = 10 and *d*_*v*_ ≥ 61.

For the remaining cases as follows:

- *s* = 1 and 4 ≤ *d*_*v*_ ≤ 12;
- *s* = 2 and 5 ≤ *d*_*v*_ ≤ 16;
- *s* = 3 and 6 ≤ *d*_*v*_ ≤ 20;
- *s* = 4 and 7 ≤ *d*_*v*_ ≤ 24;
- *s* = 5 and 8 ≤ *d*_*v*_ ≤ 29;
- *s* = 6 and 9 ≤ *d*_*v*_ ≤ 34;
- *s* = 7 and 10 ≤ *d*_*v*_ ≤ 40;
- *s* = 8 and 11 ≤ *d*_*v*_ ≤ 46;
- *s* = 9 and 12 ≤ *d*_*v*_ ≤ 53;
- *s* = 10 and 13 ≤ *d*_*v*_ ≤ 60,

we would turn to use (24), and negative upper bounds, equivalently *ABC*(*T*_1_) < *ABC*(*T*), follow from direct calculation easily.

**Case 2.** *t* = 1.

In this case, note that 0 ≤ *s* ≤ 9, and *d*_*v*_ ≥ *s* + 4 from Proposition 2.

By direct calculation, we may deduce that the right-hand side of (23) is negative, equivalently *ABC*(*T*_1_) < *ABC*(*T*), holds for the following cases:

- *s* = 1 and *d*_*v*_ ≥ 46;
- *s* = 2 and *d*_*v*_ ≥ 38;
- *s* = 3 and *d*_*v*_ ≥ 39;
- *s* = 4 and *d*_*v*_ ≥ 43;
- *s* = 5 and *d*_*v*_ ≥ 49;
- *s* = 6 and *d*_*v*_ ≥ 55;
- *s* = 7 and *d*_*v*_ ≥ 61;
- *s* = 8 and *d*_*v*_ ≥ 68;
- *s* = 9 and *d*_*v*_ ≥ 76.

For the remaining cases as follows:

- *s* = 1 and 5 ≤ *d*_*v*_ ≤ 45;
- *s* = 2 and 6 ≤ *d*_*v*_ ≤ 37;
- *s* = 3 and 7 ≤ *d*_*v*_ ≤ 38;
- *s* = 4 and 8 ≤ *d*_*v*_ ≤ 42;
- *s* = 5 and 9 ≤ *d*_*v*_ ≤ 48;
- *s* = 6 and 10 ≤ *d*_*v*_ ≤ 54;
- *s* = 7 and 11 ≤ *d*_*v*_ ≤ 60;
- *s* = 8 and 12 ≤ *d*_*v*_ ≤ 67;
- *s* = 9 and 13 ≤ *d*_*v*_ ≤ 75,

we would turn to use (24), and negative upper bounds, equivalently *ABC*(*T*_1_) < *ABC*(*T*), follow from direct calculation easily.

**Case 3.** *t* = 2.

In this case, note that 0 ≤ *s* ≤ 8, and *d*_*v*_ ≥ *s* + 5 from Proposition 2.

On one hand, the contradiction for *s* = 0 follows from Proposition 25.

On the other hand, by direct calculation, we may deduce that the right-hand side of (23) is negative, equivalently *ABC*(*T*_1_) < *ABC*(*T*), holds for the following cases:

- *s* = 1 and *d*_*v*_ ≥ 1402;
- *s* = 2 and *d*_*v*_ ≥ 107;
- *s* = 3 and *d*_*v*_ ≥ 84;
- *s* = 4 and *d*_*v*_ ≥ 81;
- *s* = 5 and *d*_*v*_ ≥ 84;
- *s* = 6 and *d*_*v*_ ≥ 89;
- *s* = 7 and *d*_*v*_ ≥ 96;
- *s* = 8 and *d*_*v*_ ≥ 104.

For the remaining cases as follows:

- *s* = 1 and 6 ≤ *d*_*v*_ ≤ 1401;
- *s* = 2 and 7 ≤ *d*_*v*_ ≤ 106;
- *s* = 3 and 8 ≤ *d*_*v*_ ≤ 83;
- *s* = 4 and 9 ≤ *d*_*v*_ ≤ 80;
- *s* = 5 and 10 ≤ *d*_*v*_ ≤ 83;
- *s* = 6 and 11 ≤ *d*_*v*_ ≤ 88;
- *s* = 7 and 12 ≤ *d*_*v*_ ≤ 95;
- *s* = 8 and 13 ≤ *d*_*v*_ ≤ 103,

we would turn to use (24), and negative upper bounds, equivalently *ABC*(*T*_1_) < *ABC*(*T*), follow from direct calculation easily.

**Case 4.** *t* = 3.

In this case, note that 0 ≤ *s* ≤ 7, and *d*_*v*_ ≥ *s* + 6 from Proposition 2.

On one hand, the contradiction for *s* = 1, 2 follows from Proposition 25.

On the other hand, by direct calculation, we may deduce that the right-hand side of (23) is negative, equivalently *ABC*(*T*_1_) < *ABC*(*T*), holds for the following cases:

- *s* = 3 and *d*_*v*_ ≥ 290;
- *s* = 4 and *d*_*v*_ ≥ 193;
- *s* = 5 and *d*_*v*_ ≥ 170;
- *s* = 6 and *d*_*v*_ ≥ 163;
- *s* = 7 and *d*_*v*_ ≥ 165.

For the remaining cases as follows:

- *s* = 3 and 9 ≤ *d*_*v*_ ≤ 289;
- *s* = 4 and 10 ≤ *d*_*v*_ ≤ 192;
- *s* = 5 and 11 ≤ *d*_*v*_ ≤ 169;
- *s* = 6 and 12 ≤ *d*_*v*_ ≤ 162;
- *s* = 7 and 13 ≤ *d*_*v*_ ≤ 164,

we would turn to use (24), and negative upper bounds, equivalently *ABC*(*T*_1_) < *ABC*(*T*), follow from direct calculation easily.

**Case 5.** *t* = 4.

In this case, note that 0 ≤ *s* ≤ 6. The contradiction may be deduced from Proposition 25.

**Case 6.** *t* = 5.

In this case, note that 0 ≤ *s* ≤ 5. The contradiction for the cases that *s* = 0 and *s* = 1, 2, 3, 4, 5 may be deduced from Propositions 24 and 25, respectively.

**Case 7.** *t* = 6.

In this case, note that 0 ≤ *s* ≤ 4. The contradiction for the cases that *s* = 0 and *s* = 1, 2, 3, 4 may be deduced from Propositions 24 and 25, respectively.

**Case 8.** *t* = 7.

In this case, note that 0 ≤ *s* ≤ 3. The contradiction for the cases that *s* = 0 and *s* = 1, 2, 3 may be deduced from Propositions 24 and 25, respectively.

**Case 9.** *t* = 8.

In this case, note that *s* = 0, 1, 2. The contradiction for the cases that *s* = 0 and *s* = 1, 2 may be deduced from Propositions 24 and 25, respectively.

**Case 10.** *t* = 9.

In this case, note that *s* = 0, 1. The contradiction for the cases that *s* = 0 and *s* = 1 may be deduced from Propositions 24 and 25, respectively.

**Case 11.** *t* = 10.

In this case, note that *s* = 0. The contradiction may be deduced from Proposition 24 directly.

Combining the above arguments, the result finally follows.

## Discussion

The characterization of minimal-ABC trees is a rather active topic in chemical graph theory these years, which has led to a lot of structural properties and potential conjectures.

It is known that every pendent vertex of minimal-ABC trees belongs to some *B*_*k*_-branch. As a strengthening, this paper proves that a minimal-ABC tree contains at most two *B*_1_-branches. Moreover, we claim that a minimal-ABC tree can not contain two *B*_1_-branches simultaneously, except for two cases that *s* = 0, and *t* = 1 or 3.

During the investigation of this paper, we also considered the two unsolved cases. However, to the best of our knowledge, until now we only get a solution under some particular degree conditions. In future research, the key point is to construct a more perfect graph transformation involve in general cases, which lead to a desired solution finally.

Actually, our ultimate goal is to show that the minimal-ABC trees contain no *B*_1_-branch, when the order of that tree is large sufficiently.
